# Analysis of deformation and failure mechanism of sandwich beams with lattice core under three-point bending load

**DOI:** 10.1038/s41598-024-64198-y

**Published:** 2024-06-10

**Authors:** Mahdi Sefidi, Hossein Taghipoor

**Affiliations:** 1 Department of Research, Design, and Product Development, Iran Khodro Co., Tehran, Iran; 2 Department of Mechanical Engineering, Velayat University, Iranshahr, Iran

**Keywords:** Sandwich beams, Energy absorption, Three-point bending, Quasi-static, Failure mechanism, Aerospace engineering, Mechanical engineering

## Abstract

This paper investigates sandwich beams with lattice cores under quasi-static bending, owing to their lightweight nature and high energy absorption capabilities. Utilizing analytical methods governing beams, an investigation into their failure mechanisms is conducted, incorporating experimental and numerical results. The influence of thickness and core cell sizes on energy absorption are examined. The analysis delves into the elastic and plastic behavior of the beam, which is refined and validated against the numerical and experimental tests and failure modes of sandwich panel beams. The alignment of analytical predictions with both experimental and numerical results in terms of mean forces, and energy absorptions was remarkably precise. Moreover, evidence has been presented that the face yield and core shear failure regions are significantly impacted by variances in core dimensions. Additionally, the thickness of core cell strands was found to be pivotal in influencing the compressive and shear strengths of sandwich panel beams.

## Introduction

Sandwich beams have been used in many constriction frames namely, cranes, bridges and ship body structures especially in transportation systems. The mentioned beams are made as one assembly process or more and their cores are units. Also, they are be able to withstand a broad range of dynamic loads and forces are caused by sea waves and winds. Due to their regular and periodic core of these structures, they can absorb energy. Besides, due to their low weight, high strength and type geometric of cores, they have an acceptable stiffness against the shear forces^1,2^. Hexagonal honeycomb structures with polymer foam are one of the most applicable sandwich panels which are used in the different industries as energy absorption. Foam Cores have many applicable because of some properties such as insulation against noise and water penetration and heat resistance^3^. Besides, Due to the production cost has been played a significant role in production of each structure hence, using these polymer foams can be more affordable than other cores^4^.

A constant response of the force–displacement, along with maximum energy absorption, is deemed the ideal mechanical response for an energy-absorbing system^5^. Due to unique advantages of the metal sandwich structures with cellular cores or lattice material cores which are related to their high strength and absorption rate, they are implemented as absorber in airplanes, spacecraft and some cars which need light weight. Besides, in these types of structures, the required stiffness have been provided by the upper and lower sheets of the sandwich beams^6–9^. In some cases, for increasing the bending and shear strength have been used two-dimensional design lattices structures and foams as core in sandwich beams because they have an acceptable energy absorption rate^10^.

In recent years, Designing substructures to counter the explosion effects and the effects of shock forces has become essential^11^. There are many types of the core topologies in sandwich panels which one the most popular of them is the expanded sheet metal core. These cores have been used as a structure to decrease the level of explosive forces and penetration by their stiffness and strength in both vertical and longitudinal directions^12,13^. The axial crushing and transvers bending responses of the sandwich structures with the lattice core has been presented by Taghipoor et al.^14^. The cell size and cell orientation effects on the energy absorption have been investigated and they found that the deformation mode of sandwich beams under the three-point dynamic load were the same as the quasi-static load and the major difference between them was related to their peak forces. According to their results, the number of layer core and the cell size are the main key factors for these beams in the quasi-static tests^15^.

Using of Expanded metal as core in the sandwich beam for improving the performance and energy absorption rate has been growing in recent years^16^. Hence, many works has been taken to mix the cellular cores and the expanded sheet metal cores with the polymer foam to rise the energy absorption trend^17^. The study by Taghipoor et al.^18^ explores the energy absorption potential of sandwich beams, employing rigid polyurethane foam and expanded metal sheets as the core, through bending tests. Results demonstrate substantial enhancements in energy absorption up to 80% with foam reinforcement and 74.6% by optimizing lattice sheet orientation. Kooistra et al.^19^ propose a novel method for crafting metallic lattice truss structures, utilizing a pyramidal lattice design to bolster node robustness and optimize sheet material utilization. Aluminum alloy lattices, engineered with a relative density of 5.7%, demonstrate exceptional performance in compression, transverse, and longitudinal shear tests, closely mirroring theoretical predictions and exhibiting no node failures even under substantial plastic shear straining. In this study by Jafari Nedoushan^20^, various methods to enhance the crashworthiness of expanded metal tubes were examined through experimental and numerical analyses. Techniques such as stiffening, foam filling, and multi-layer configurations were evaluated, with results showing the significant influence of cell size on crashworthiness. Improved energy absorption properties were observed, particularly in foam-filled tubes, with finite element modeling accurately predicting load–displacement responses and facilitating further parameter investigation. This study contributed to the understanding of crashworthiness enhancement strategies for expanded metal tubes. In this investigation by Smith^21^, the structural behavior of flattened expanded metal tubes under axial crushing is explored. Experimental analysis initially examines the impact of the angle between the expanded metal cell and the applied load, comparing results with standard expanded metal sheets. Subsequently, nonlinear finite element models are employed for numerical analyses, revealing enhanced energy absorption characteristics due to tube flattening. Both experimental and numerical findings demonstrate a notable increase in energy absorption capacity and mean force for the flattened tubes.

In a research taken by Li et al.^22^ they presented an elastic–plastic model to determine the behavior of composite sandwich beams under the dynamic load. Also, they investigated the effective characterizes of the bending in three regimes, namely the elastic, core-crushing and final failure regime. Evans et al.^23^ compared a type of stochastic cellular materials with periodic cells and configured as cores of panels, tubes, and shells then they found that the mechanical properties and segment performance of these type of cores have been improved. Xiang et al.^24^ studied on the behavior of the sandwich beams with thin-walled tubes core which have been under the three-point bending. They determined an analytical relation between the force and displacement at the mid-span of the sandwich beam integrated by experiment tests. Han et al.^25–27^ studied the collapse mechanism of a sandwich panel which improved with an aluminum foam-reinforced corrugated core under the quasi-static and dynamic load with different strain rates. The obtained results of the foam-free cases presented that the corrugated plates were dominated by bending deformation also, they collapsed easily. Besides, the beneficial effect of foam filling has been demonstrated in the strength and energy absorption and for the much larger values of the slenderness ratio, the foam filling effects have been decreases gradually. Alavi Nia and their colleagues^28^ accomplished some experimental studies on the mechanical behavior of the hollow honeycomb filled with polyurethane foam under the compressive load. Xiong et al.^29^ obtained different failure modes and mechanical properties of panels with different sizes. In general, the measured failure loads of the experiment tests were close to the theoretical values. The effect of using the polypropylene foam in aluminum honeycomb panels have been calculated by Liu et al.^30^ and they found that the foam reinforcement had no significant effect on its deformation pattern under axial crushing, whereas it helped to reduce local compression in honeycomb panels. Vaidya et al.^31^ measured the response of sandwich steel beams with corrugated cores under the quasi-static load. The core arrangement and the beam span have been found as key factors in the quasi-static load.

The majority of prior works have focused on the lattice core structure, and a few limited studies have been performed on the sandwich beams with expanded metal sheet cores. Besides, the most experiments have been performed by axial quasi-static load and a few studies investigated the three-point bending on the sandwich panels with the lattice core. In this study, considering that the influence of thickness and core cell sizes on energy absorption is achieved through plastic deformation in beams, the governing equations for plastic hinges within the structure are employed. For predicting the failure mode maps of the sandwich beams with lattice core, the Gibson model is utilized. Furthermore, strain energy density is employed to determine the critical compressive stress and elastic stiffness.

Hence, in the present study, the mechanical behavior of sandwich beams with an expanded metal sheet cores under a three-bending quasi-static load are investigated as the analytical method. Also, the effect of the core geometric parameters on the energy absorption and the mean force are studied. This research endeavors to provide an analytical relation for the analysis of deformation behavior and the failure mechanism of the sandwich panel beams with lattice cores under three-point bending loads by the modified elastic and plastic equations governing beams.

## Material and methods

### Metal core

**Figure 1:**
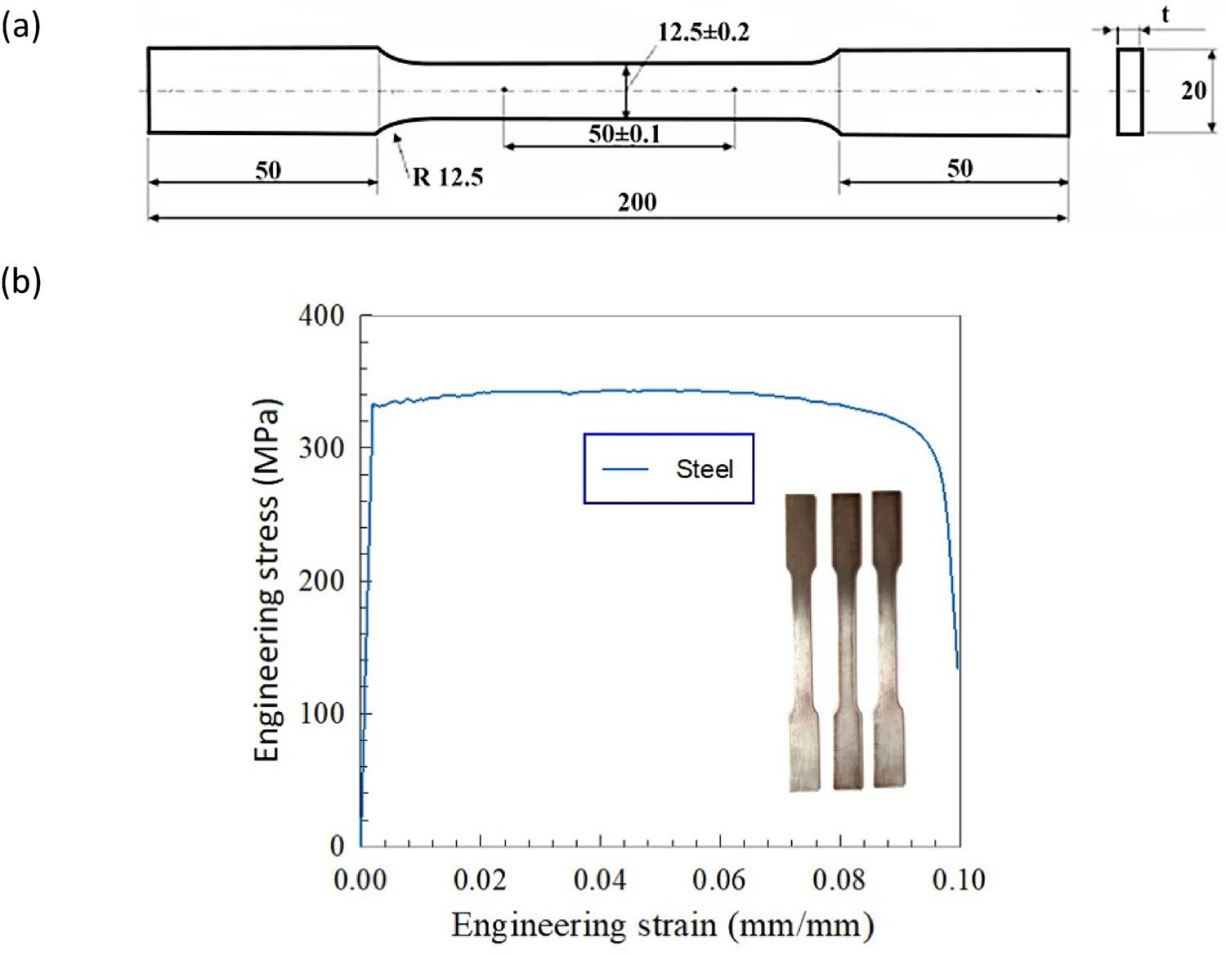
Details of tensile test for steel sheet; (**a**) ASTM E08M standard details (Dimensions in *mm*), (**b**) Engineering stress–strain curve^15^.

The expanded metal sheets were fabricated using a cold-rolled ASTM A-611 high-strength steel^15^. Standard tensile strength test was performed according to ASTM E08M-04^32–35^ on three standard samples directly cut from the sheets (as shown in Fig. 1a) in order to evaluate the material characteristics of the steel sheets. In Fig. 1b, the engineering stress versus engineering strain of the steel sheets under tensile test is presented. Also, the obtained results are listed in Table 1.

**Table 1:** Mechanical properties of steel.

Material	*ρ* (kg/m3)	*E* (GPa)	*ν*	*σ*_*y*_ (MPa)	*σ*_*u*_ (MPa)
Steel	7800	201	0.3	333.5	363.5

### Core fabrication

**Figure 2:**
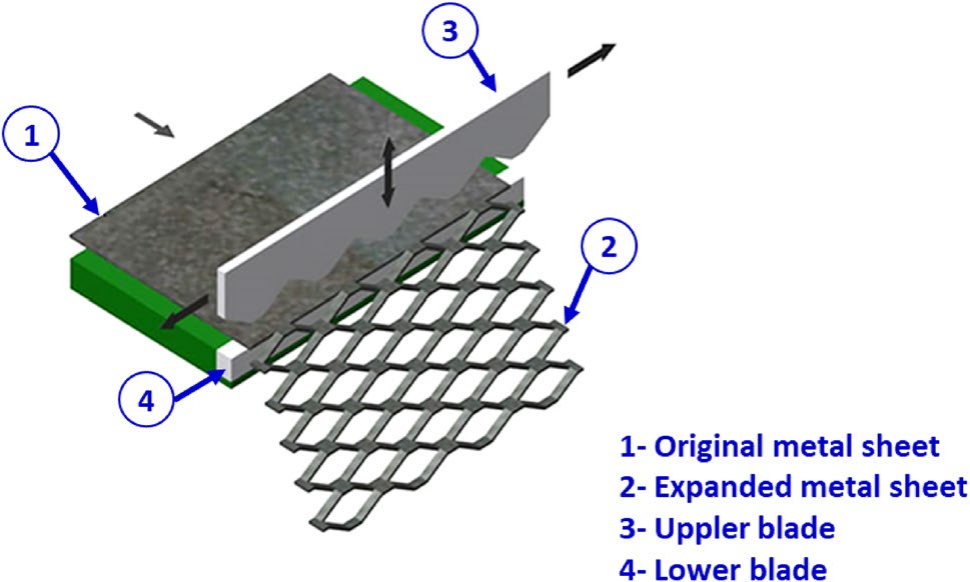
Schematic of the slitting and expanding process—raised expanded metal sheets^36^.

The production of expanded metal mesh, initially known as slashed metallic screening, involves the simultaneous slitting and stretching of a metal sheet, producing a diamond-shaped pattern (Fig. 2). This process improves the material’s strength-to-weight ratio, rendering it well-suited for sandwich panel structures across various applications. Expanded metal sheets are routinely fabricated in two basic types: standard expanded metal (SEM) and flattened expanded metal^36^.

**Figure 3:**
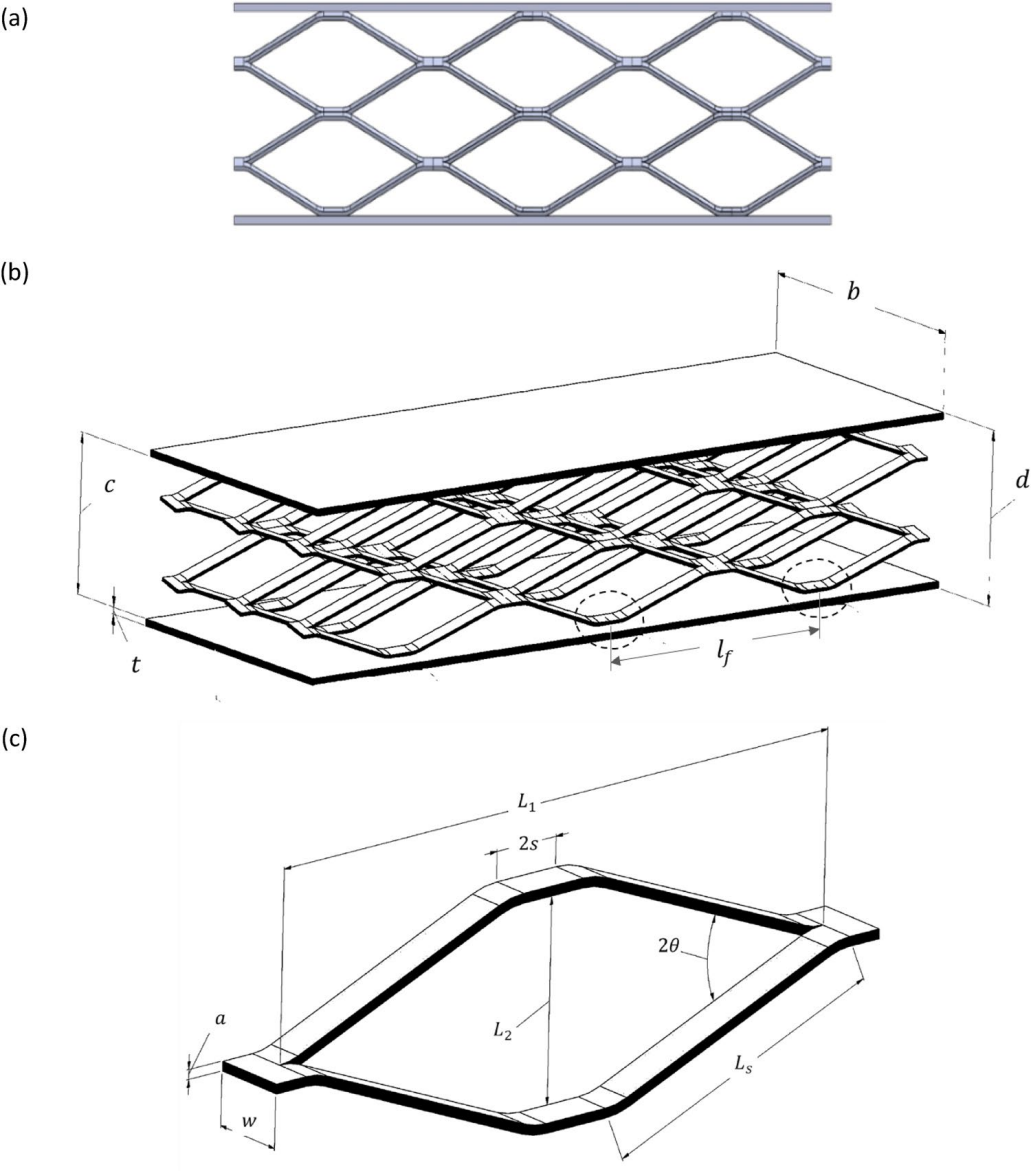
Schematic view of; (**a**) configuration of sandwich beams (Horizontally oriented), (**b**) sample (C332), (**c**) an expanded metal cell.

In this research, SEM has been employed and the sandwich beams were configured with multiple diverse cores, and bonded to two thick face sheets, where, thickness of $t,$ width $b$, with $c$, core height, $d$ is panel height, and ${l}_{f}$ is the spacing between the connection point of core cells and the face-sheet, which depicted in Fig. 3a,b. The geometry of the pattern is mainly characterized by two orthogonal axes, which ${L}_{1}$ is the major axis and ${L}_{2}$ the minor, ${L}_{s}$ is strand length, a is the strand thickness, 2 s is the spacing between strands, and w is the strand width, as shown in Fig. 3c. For preparation of the sandwich beams, acetylene welding is used to connect the expanded metal sheet core to the top and bottom substrates.

### Analytical models

In order to achieve an analytical model for energy absorption, the elastic and plastic behavior of a sandwich beam under three-point bending has been employed. To determine the average force and plastic behavior of the beam, the identification of locations forming plastic hinges has been scrutinized. Since the joints and nodes formed in each cell of the expanded metal mesh act as a plastic hinge in the collapse mechanism, they participate in energy absorption. Subsequently, an analytical method for predicting collapse states for a sandwich beam with an expanded metal mesh core under flexural loading is presented.

#### Elastic bending deformation of sandwich beams

**Figure 4:**
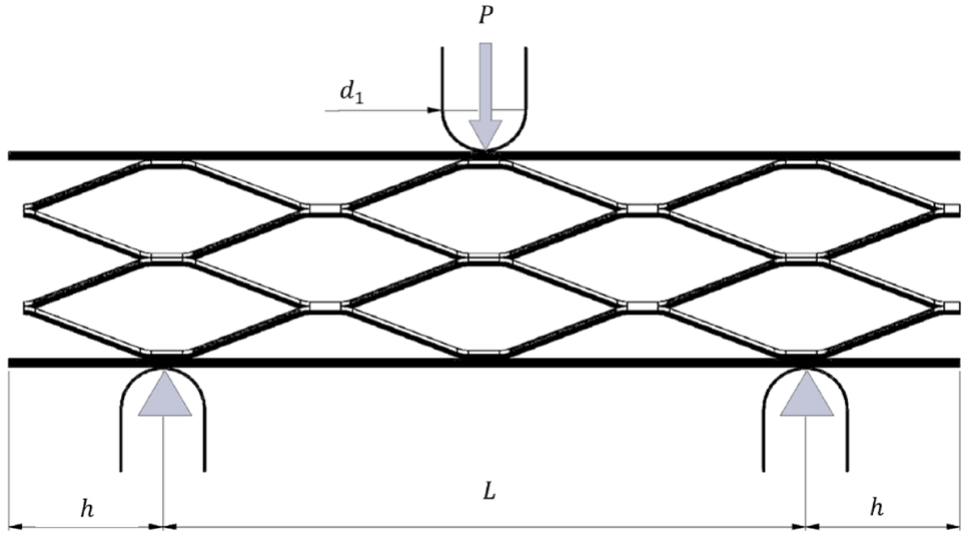
A perspective of sandwich beam under three-point bending load.

Considering a beam consisting of two face-sheets with a thickness of $t$, length $L$, width $b$, Overhang distance beyond the support $h$, the width of the loading head is ${d}_{1}$ , and $c$ is core thickness, subjected to a bending load $P$ (Fig. 4), the deflection of the beam $\delta$, equivalent flexural stiffness ${(EI)}_{eq}$, and equivalent shear stiffness ${(AG)}_{eq}$ can be calculated according to Eqs. (1–3)^37,38^:

1 $$ \delta = \frac{{P \times L^{3} }}{{48 \times \left( {E \times I} \right)_{eq} }} + \frac{P \times L}{{4 \times \left( {A \times G} \right)_{eq} }} $$

2 $$ \left( {EI} \right)_{eq} = \frac{{E_{s} \times b \times d \times t^{2} }}{2} + \frac{{E_{s} \times b \times t^{3} }}{6} + \frac{{E_{c} \times b \times c^{3} }}{12} \approx \frac{{E_{s} \times b \times d \times t^{2} }}{2} $$

3 $$ \left( {AG} \right)_{eq} = \frac{{b \times \left( {c + t} \right)^{2} \times G_{c} }}{c} \approx b \times c \times G_{c} $$

where ${E}_{s}$ is the young’s modulus of the face sheet, ${E}_{c}$ is the young’s modulus of the core, ${G}_{c}$ is the shear modulus of core and $I$ is the moment of inertia about the horizontal centroidal axis (the neutral axis). Moreover, in Eq. (3), the face-sheets are assumed much thinner than the core also, the face sheet modulus is much greater than the core.

#### Elastic stiffness

**Figure 5:**
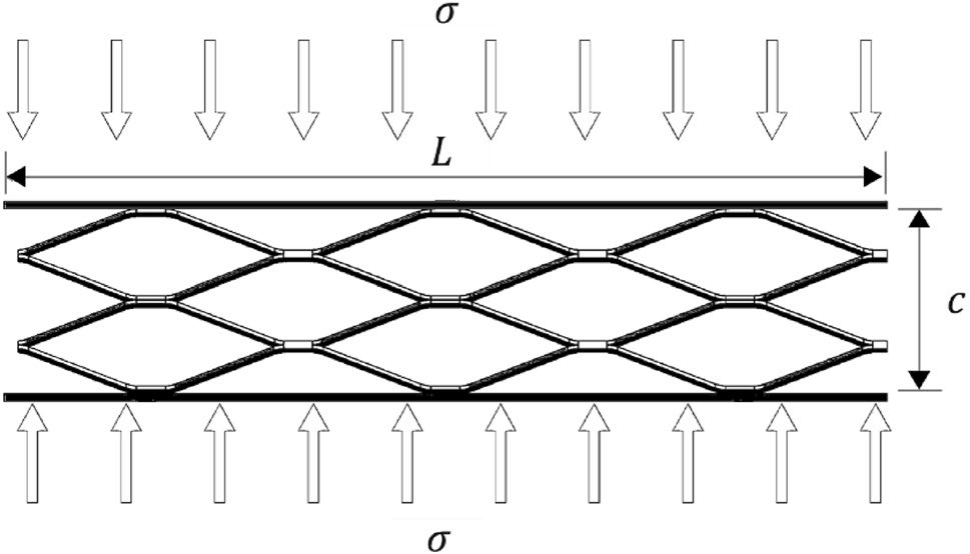
Sketch of a sandwich plane under out-of-plane compression.

To obtain the modulus of elasticity in the core of a sandwich beam, equations for the strain energy generated at the joints with cross-sectional area “$wa/2$” under out-of-plane compression, according to Fig. 5, are written as follow^39^:

4 $$ \frac{1}{2} \times \frac{{\sigma^{2} }}{{E_{c} }} \times L \times c \times w = \frac{1}{2} \times \frac{{\sigma_{{y \left( {core} \right)}}^{2} }}{{E_{s} }} \times N_{j} \times \frac{wa}{2} \times \left( {L_{s} + 2s} \right) $$

where ${{\sigma }_{y}}_{(core)}$ is stress in joints of the core, ${N}_{j}$ is total joints of the core. Also, the equilibrium about the joint gives:

5 $$ \sigma \times L \times b = \sigma_{{y_{{\left( {core} \right)}} }} \times N_{j} \times a \times \frac{b}{2} $$

Equilibrium in the out-of-plane direction gives the relation between the stress $\sigma$ and the maximum compressive stress of a core ${{\sigma }_{y}}_{(core)}$ as:

6 $$ \sigma = \sigma_{{y_{{\left( {core} \right)}} }} \times \frac{{N_{j} }}{2} \times \frac{a}{L} $$

Substituting Eq. (6) into Eq. (4) the elastic modulus of the core is given by:

7 $$ \frac{{E_{c} }}{{E_{s} }} = \frac{{N_{j} }}{2} \times \frac{c}{L} \times \frac{a}{{\left( {L_{s} + 2s} \right)}} $$

Furthermore, to calculate the core density of sandwich panels, the following relationships are derived based on the volume fraction of the unit cell and member as^19^:

8 $$ \overline{\rho } = \frac{{V_{T} }}{{V_{C} }} = \frac{{4 \times L_{s} \times a \times \frac{w}{2} + 4 \times a \times s \times w}}{{w \times \left( {2L_{s} \times \sin \theta + 2a} \right) \times \left( {2L_{s} \times \cos \theta + 4s} \right)}} $$

9 $$ \overline{\rho } = \frac{{a \times \left( {L_{s} + 2s} \right)}}{{\left( {L_{s} \times \sin \theta + a} \right) \times \left( {L_{s} \times \cos \theta + 2s} \right)}} $$

where ${V}_{T}$ and ${V}_{C}$, the unit cell volumes and member volumes. By utilizing Eq. (9) and substituting it into Eq. (7), the following relations are derived:

10 $$ \frac{{E_{c} }}{{E_{s} }} = \frac{{N_{j} }}{2} \times \frac{{a^{2} }}{L} \times \frac{c}{{\overline{\rho } \times \left( {L_{s} \times \sin \theta + a} \right) \times \left( {L_{s} \times \cos \theta + 2s} \right)}} $$

#### Shear stiffness

**Figure 6:**
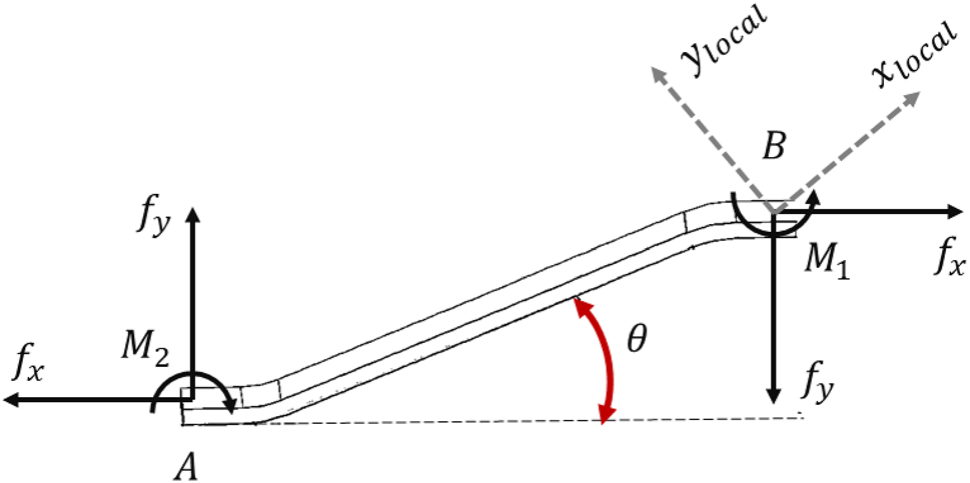
Free-body diagram of member of core cell.

Due to the uniform force experienced by the four members of the unit cell, an estimation of the nominal strength of the corrugated cores can be made by examining the deformation of a single member, illustrated in Fig. 6.

In the direction of $x$, the force ${f}_{x}$ is exerted, and along the $y$-axis, it is met with the reactive force ${f}_{y}$. Additionally, moments ${M}_{1}$ and ${M}_{2}$ act at two levels, “A” and “B”, respectively. By taking moments around point “A”, the calculation is:

11 $$ \sum M_{A} = 0 \to f_{y} \times L_{s} \times cos\theta + f_{x} \times L_{s} \times sin\theta - M_{1} - M_{2} = $$

To calculate the shear stiffness, vertical deformations are assumed to be zero around point “B” ($\Delta y=0$):

12 $$ \frac{{L_{s} }}{A} \times \frac{{\sin \theta \times \left( {f_{x} \times \cos \theta - f_{y} \times \sin \theta } \right)}}{{E_{s} }} + \left[ {\frac{{\left( {f_{y} \times \cos \theta - f_{x} \times \sin \theta } \right) \times L_{s}^{3} }}{{3 \times E_{s} \times I}} - \frac{{M_{1} \times L_{s}^{2} }}{{2 \times E_{s} \times I}}} \right] \times \cos \theta = 0 $$

Furthermore, the gradient at the hinge about point B is equal to zero ($\dot{\Delta }y=0$); consequently, it is established that:

13 $$ - \frac{{M_{1} \times L_{s} }}{{E_{s} \times I}} + \left[ {\frac{{L_{s}^{2} }}{{E_{s} }} \times \frac{{\left( {f_{y} \times \cos \theta + f_{x} \times \sin \theta } \right)}}{2 \times I}} \right] = 0 $$

Utilizing Eqs. (11–13), the forces and moments are equated as follows:

14 $$ f_{y} = f_{x} \times \frac{{\left( {12 \times I - A \times L_{s}^{2} } \right)}}{{12 \times I \times sin^{2} \theta + A \times L_{s}^{2} \times cos^{2} \theta }} \times \sin \theta \times \cos \theta $$

15 $$ M_{1} = M_{2} = f_{x} \times L_{s} \times \frac{6 \times I \times sin\theta }{{12 \times I \times sin^{2} \theta + A \times L_{s}^{2} \times cos^{2} \theta }} $$

The force in ${x}_{local}$ is defined as:

16 $$ f_{{x_{local} }} = f_{x} \times cos\theta - f_{y} \times sin\theta $$

17 $$ f_{{x_{local} }} = \frac{{A \times L_{s}^{2} \times cos\theta }}{{12 \times I \times sin^{2} \theta + A \times L_{s}^{2} \times cos^{2} \theta }} \times f_{x} $$

The displacement in the direction $x$ is defined as:

18 $$ \delta_{x} = \frac{{f_{{x_{local} }} \times L_{s} }}{{E_{s} \times A}} \times \cos \theta = \frac{{f_{x} }}{{E_{s} }} \times L_{s} \times \left( {\frac{{L_{s}^{2} \times \cos^{2} \theta }}{{12 \times I \times \sin^{2} \theta + A \times L_{s}^{2} \times \cos^{2} \theta }}} \right) $$

By using Eq. (18), the shear strain can be obtained as:

19 $$ \gamma_{yx} = \frac{{\delta_{x} }}{{L_{s} \times \sin \theta }} = \frac{{f_{x} }}{{E_{s} }} \times \frac{{L_{s}^{2} }}{{\left( {12 \times I \times \sin^{2} \theta + A \times L_{s}^{2} \times \cos^{2} \theta } \right)}} \times \frac{{\cos^{2} \theta }}{\sin \theta } $$

To calculate shear stress on the $xy$-plane:

20 $$ \tau_{yx} = \frac{{4 \times f_{x} }}{{A_{c} }} $$

where ${A}_{c}$ is the area over which the force is applied on the $xz$-plane and for core with horizontally oriented, it is equal to:

21 $$ A_{core} = A_{c} = w\left( {2L_{s} cos\theta + 4s} \right) $$

Substituting calculated formulas into the shear modulus equation, it can be expressed:

22 $$ G_{c} = \frac{{\tau_{yx} }}{{\gamma_{yx} }} = \frac{2a}{{L_{s}^{2} }} \times \frac{w}{{A_{c} }} \times \frac{\sin \theta }{{\cos^{2} \theta }} \times E_{s} \times \left( {4 \times a^{2} \times \sin^{2} \theta + L_{s}^{2} \times \cos^{2} \theta } \right) $$

The maximum shear stress ($G_{c}$), which arises due to the buckling of strand in the core cell, occurs when the axial force applied to the strands equates to the critical buckling force ($P_{cr}$) on a cell. Hence, it can be presented as:

23 $$ P_{{cr_{{\left( {core} \right)}} }} = \pi^{2} \times E_{s} \times \frac{I}{{L_{s}^{2} }} $$

Given that buckling occurs around an axis of the cross-section where the moment is minimal, the moment of inertia equal to:

24 $$ I = \frac{1}{12} \times \left( \frac{w}{2} \right) \times a^{3} $$

Therefore, substituting Eqs. (23) and (24) into (17), the maximum force in the x-direction (${f}_{x}$) and the critical shear strength of the core (${{\tau }_{y}}_{(core)}$) for buckling failure mode is given by:

25 $$ f_{x} = \frac{{12 \times I \times \sin^{2} \theta + A \times L_{s}^{2} \times \cos^{2} \theta }}{{A \times L_{s}^{2} \times \cos \theta }} \times P_{{cr_{{\left( {core} \right)}} }} $$

26 $$ \tau_{{y_{{\left( {core} \right)}} }} = \frac{{2 \times \pi^{2} \times a^{3} }}{{3 \times L_{s}^{4} }} \times \frac{w}{{A_{c} }} \times \frac{{E_{s} \times \left( {a^{2} \times \sin^{2} \theta + L_{s}^{2} \times \cos^{2} \theta } \right)}}{\cos \theta } $$

To compute the compressive stress, such as the procedure utilized for assessing shear stress, the displacement in the x-direction is set to zero, thus:

27 $$ \Delta x = 0 $$

28 $$ \frac{{L_{s} }}{A} \times \frac{{\cos \theta \times \left( {f_{x} \times \cos \theta - f_{y} \times \sin \theta } \right)}}{{E_{s} }} + \left[ {\frac{{\left( {f_{y} \times \cos \theta + f_{x} \times \sin \theta } \right) \times L_{s}^{3} }}{{3 \times E_{s} \times I}} - \frac{{M_{1} \times L_{s}^{2} }}{{2 \times E_{s} \times I}}} \right] \times \sin \theta = 0 $$

Due to the gradient at the hinge about point B is equal to zero ($\dot{\Delta }x=0$); it is established that:

29 $$ - \frac{{M_{1} \times L_{s} }}{{E_{s} \times I}} + \left[ {\frac{{L_{s}^{2} }}{{E_{s} }} \times \frac{{\left( {f_{y} \times \cos \theta + f_{x} \times \sin \theta } \right)}}{2 \times I}} \right] = 0 $$

Substituting Eqs. (28) and (29) into (14) and (15), the force and the moment equilibrium about the origin give:

30 $$ f_{x} = f_{y} \times \frac{{\left( {12 \times I - A \times L_{s}^{2} } \right)}}{{12 \times I \times \cos^{2} \theta + A \times L_{s}^{2} \times \sin^{2} \theta }} \times \sin \theta \times \cos \theta $$

31 $$ M_{1} = M_{2} = f_{y} \times L_{s} \times \frac{6 \times I \times \cos \theta }{{12 \times I \times \cos^{2} \theta + A \times L_{s}^{2} \times \sin^{2} \theta }} $$

Also, the force in ${x}_{local}$ is given as:

32 $$ f_{{x_{local} }} = \frac{{A \times L_{s}^{2} \times \sin \theta }}{{12 \times I \times \cos^{2} \theta + A \times L_{s}^{2} \times \sin^{2} \theta }} \times f_{y} $$

Such as the procedure utilized for assessing shear stress, the critical compressive stress of the core ($\sigma_{{y_{{\left( {core} \right)}} }}$) is given as:

33 $$ \sigma_{{y_{{\left( {core} \right)}} }} = \frac{{2 \times \pi^{2} \times a^{3} }}{{3 \times L_{s}^{4} }} \times \frac{w}{{A_{c} }} \times \frac{{E_{s} \times \left( {a^{2} \times \cos^{2} \theta + L_{s}^{2} \times \sin^{2} \theta } \right)}}{\sin \theta } $$

#### Plastic bending deformation of sandwich beams

**Figure 7:**
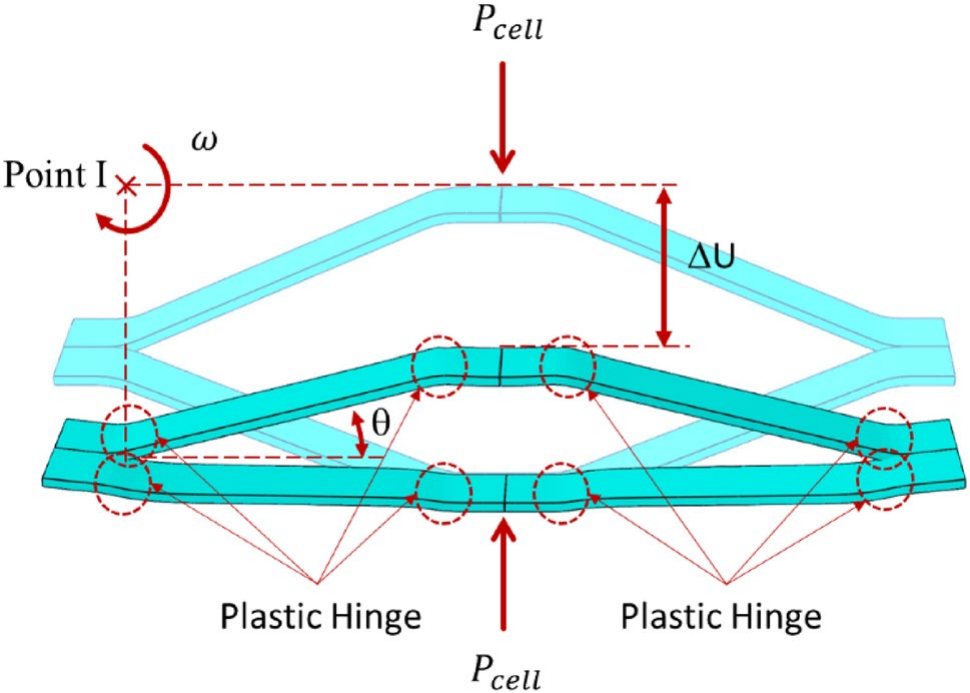
Plastic deformation mechanism of one cell and plastic hinges after taken load.

Applying the force of ${P}_{cell}$ to a cell with a large strand length of ${L}_{1}$ (Fig. 3c) results in the collapse of this cell, during which 8 plastic hinges are formed as depicted in Fig. 7. Assuming the assumption of the cell center, each set of four joints form a mechanism, such that around its center, they rotate instantaneously with an angular velocity of $\omega$. The load, ${P}_{cell}$, causes the two surfaces indicated in Fig. 7 to approach each other at a velocity of $\omega \frac{{L}_{1}}{2}$ . Consequently, the work done by the force ${P}_{cell}$ and plastic hinges is equal to:

34 $$ 2 \times P_{cell} \times \frac{{L_{1} }}{2} \times \omega = 8 \times M_{p} \times \omega $$

**Figure 8:**
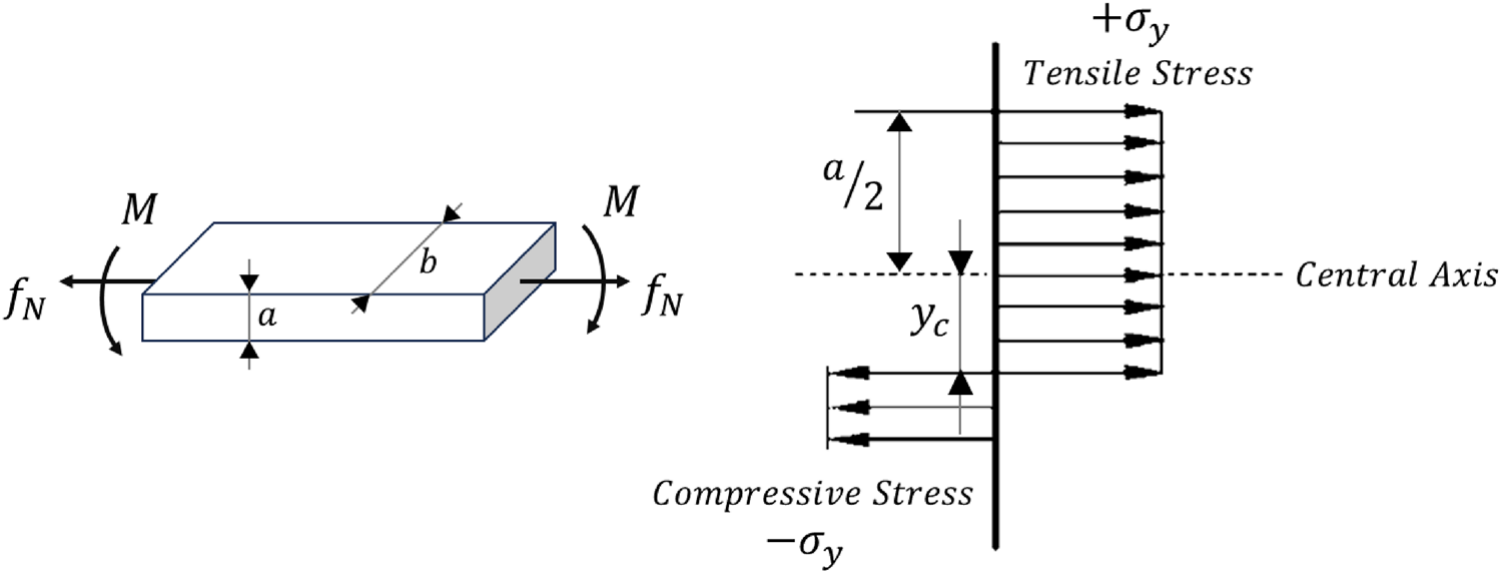
The stress distribution on a cross-section of a rectangular beam subjected to both bending moment $M$ and tensile force ${f}_{N}$^40^.

In this context, ${L}_{1}$ represents the length of the major cell strand, when the applied moment leads to yielding at the joints, a plastic hinge with a moment capacity equal to ${M}_{p}$ is made. As depicted in Fig. 8, the plastic bending moment for sections subjected solely to flexural loads occurs when the neutral axis is precisely at the center and the distance from the neutral axis (${y}_{c}$) becomes zero. In this case, considering${\sigma }_{y}$, as yield strength, the plastic moment is calculated as^40^:

35 $$ M_{p} = \sigma_{y} \times b \times \left( {\frac{a}{2} - y_{c} } \right) \times \left( {\frac{a}{2} + y_{c} } \right) = \sigma_{y} \times b \times \left( {\frac{{a^{2} }}{4} - y_{c}^{2} } \right),y_{c} = 0 \cong \sigma_{y} \times b \times \frac{{a^{2} }}{4} $$

Hence, the magnitude of the plastic moments generated in the upper and lower face sheet of a sandwich panel, ${\left({M}_{p}\right)}_{face}$, and the joints of the cells, ${\left({M}_{p}\right)}_{cell}$, respectively, are equivalent to:

36 $$ \left( {M_{p} } \right)_{face} = \sigma_{y} \times b \times \frac{{t^{2} }}{4} $$

37 $$ \left( {M_{p} } \right)_{cell} = \sigma_{y} \times w \times \frac{{a^{2} }}{4} $$

In consideration of Eqs. (34) and (37), the required force for the formation of a plastic hinge in a cell is equal to:

38 $$ P_{cell} = 2 \times \sigma_{y} \times w \times \frac{{a^{2} }}{{L_{1} }} $$

On the other hand, the level of work accomplished is equivalent to the sum of the energy involved in the deformation of the plastic core cells and face sheets, which is calculated from the following equation:

39 $$ E_{T} = E_{face} + E_{core} { } \to { }P \times U_{yb} = 2 \times \left( {M_{P} } \right)_{face} \times \alpha + \mathop \sum \limits_{i = 1}^{{N_{c} }} P_{cell} \times U_{yc} $$

**Figure 9:**
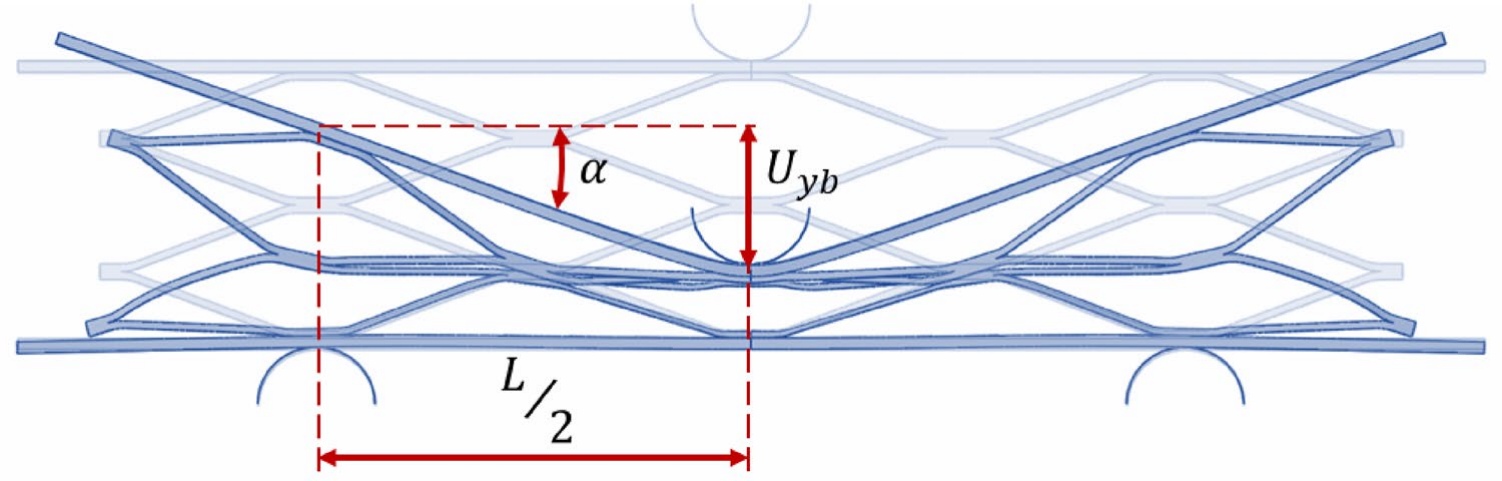
Displacement of the sandwich beam center under *P* load.

In the given context, ${N}_{c}$ represents the count of core cells, ${U}_{yb}$ represents the displacement at the center of the beam, as illustrated in Fig. 9.

Also, ${U}_{yc}$ corresponds to the deflection of the cell. The relationship between ${U}_{yb}$ and ${U}_{yc}$ is equal to:

40 $$ U_{yb} = m \times U_{yc} = \frac{L}{2} \times \alpha \to U_{yc} = \frac{L \times \alpha }{{2 \times m}} $$

where $m$ denotes the count of central cells that are highly deformed, which were subjected to complete deflection. In the analytical results, the displacement and crushing length of all cells under bending loads are assumed to be uniform.

**Figure 10:**
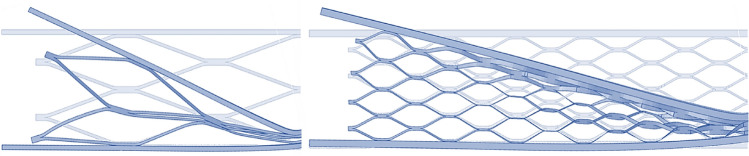
View of the collapse of two different size cores.

However, in experimental and numerical simulation results, as depicted in Fig. 10, central cells have undergone complete crushing, while cells further away from the sandwich beam’s center have experienced less crushing compared to central cells. Moreover, there exists a discrepancy among the energy absorption values obtained from experimental specimens and numerical studies, and the analytical results. To rectify this discrepancy and enhance the precision of analytical equations, the crushing behavior observed in experimental specimens and numerical simulations has been utilized to determine the parameter “${\beta }_{i}$” for each beam accordingly. Hence, by substituting Eq. (40) into Eq. (39), the dissipative force for deformation of the core and face sheet is equal to:

41 $$ P = \sigma_{y} \times b \times \frac{{t^{2} }}{L} + \mathop \sum \limits_{i = 0}^{{N_{c} }} \beta_{i} \times 2 \times \sigma_{y} \times w \times \frac{{a^{2} }}{{m \times L_{1} }} $$

where ${\beta }_{i}$, represents a coefficient indicative of the deformation magnitude of each cell relative to the central cells, with its value varying in each cell in proportion to its distance from the point of force application (${\beta }_{i}$ in central cells are equal 1). With the aid of Eq. (41), which is equivalent to the mean force depicted in the force–displacement graph, the amount of energy absorbed by the sandwich panel beam can be calculated.

**Figure 11:**
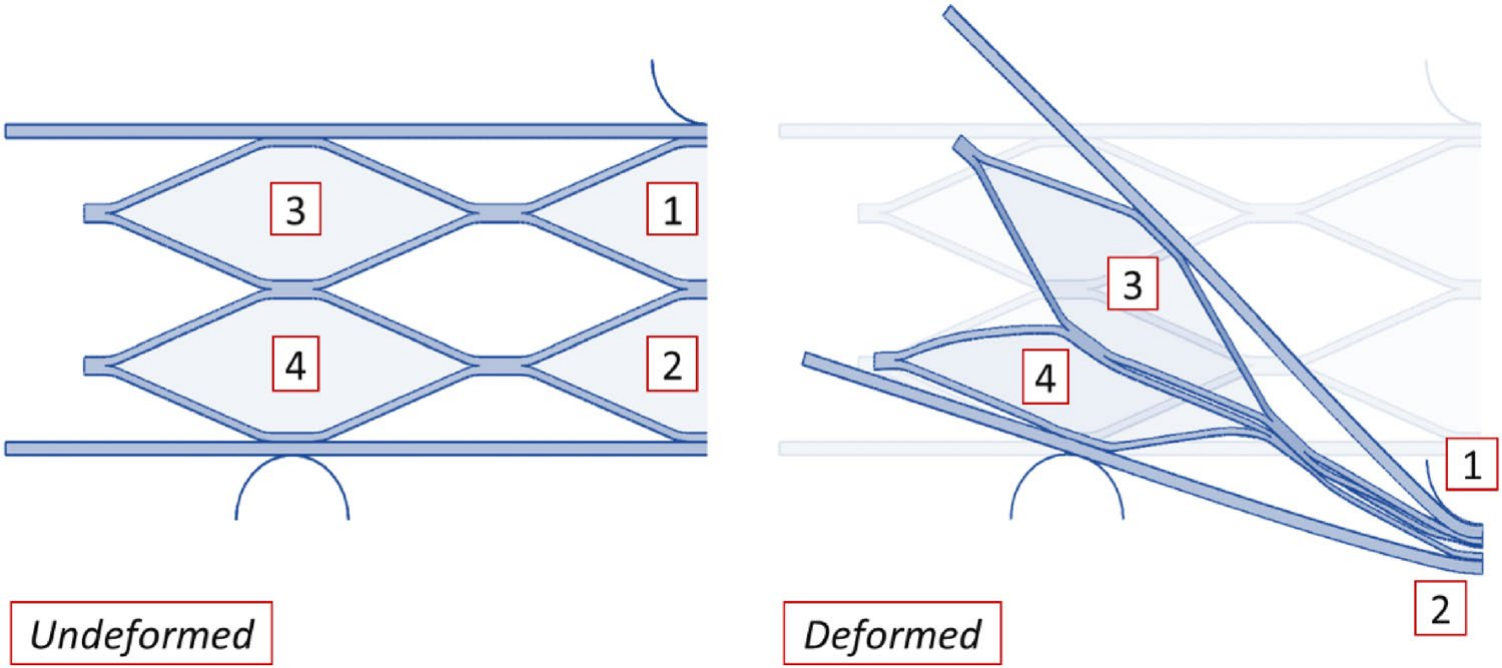
View of the collapse cores.

Given the assumption of symmetry in the core of the sandwich panel in numerical simulation^15^, as depicted in section a of Fig. 11, cells No. 1 and No. 2 have completely collapsed, while cells No. 3 and No. 4 each experienced a specific ratio of collapse. To obtain the collapse ratios of cells No. 3 and No. 4 relative to cells No. 1 and No. 2, the output results of the numerical simulation was utilized.

### Failure modes

The type of collapse in structures and the general failure of beams depend on the materials used. For instance, beams with composite face-sheets and a metallic foam core experience micro-scale buckling in the face-sheets, whereas if ceramic sheets are used for the upper and lower face-sheets, the ceramic face-sheets exhibit elastic behavior before fracturing under compressive loads, with their core, made of metallic foam, undergoing plastic failure after the beam yields^41^.

**Figure 12:**
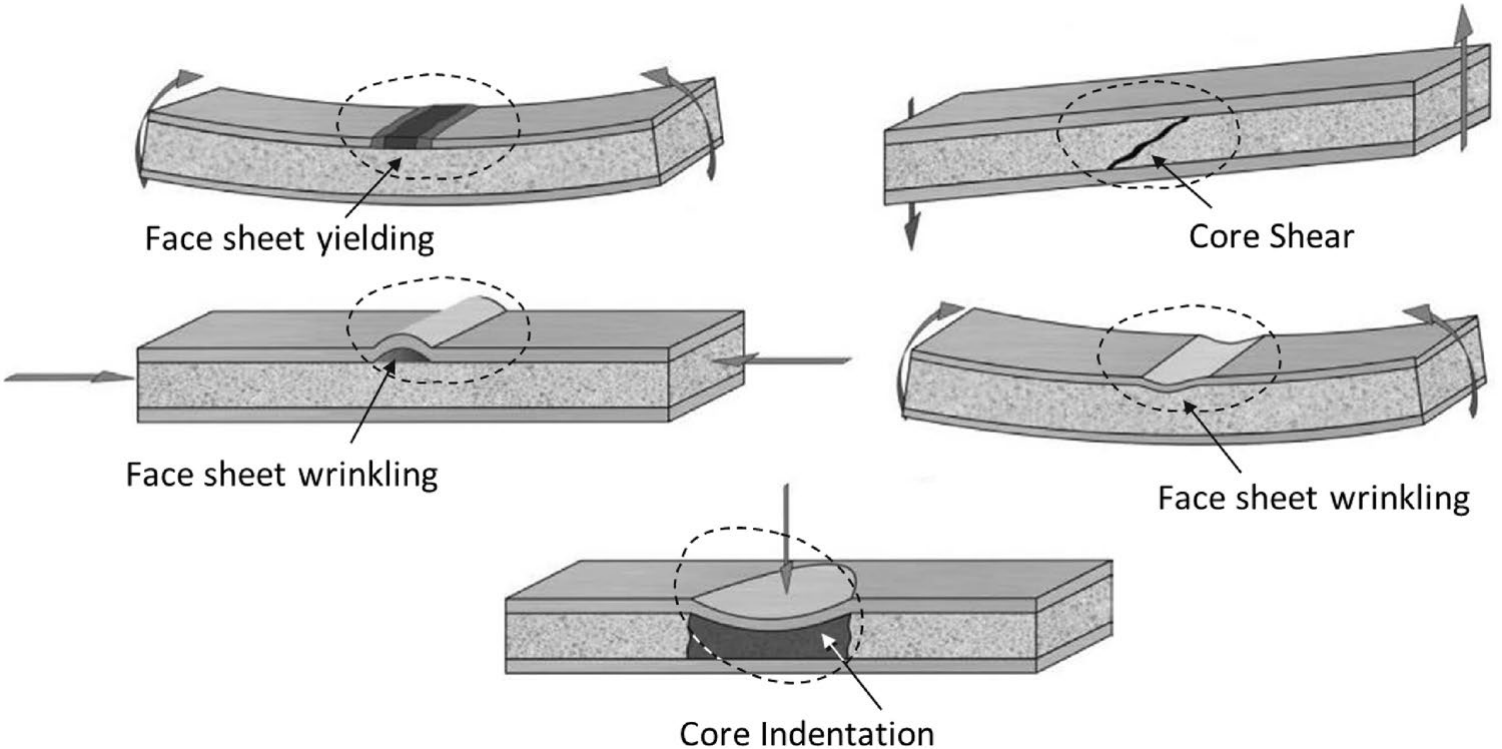
Different failure modes of sandwich structure^42^.

In the context of failure in sandwich panel beams, four main modes of failure have been identified: yielding of the face-sheets, wrinkling of the face-sheet due to sheet compression, core shear, and core failure at the impact area zone (the indentation mode), which are determined based on the maximum applied force within the elastic area (Fig. 12).

#### Face sheet yielding

Face-sheet yielding occurs when the face-sheets attain the yielding strength. The collapse yielding strength define as ${\sigma }_{{y}_{(face)}}$, and by overlooking the role of the core in contributing to bending strength in beam, ${\sigma }_{{y}_{(core)}}$, the critical load associated with the face-sheet yielding, ${P}_{{y}_{(face)}}$, is^43^:

42 $$ P_{{y_{{\left( {face} \right)}} }} = 4 \times \sigma_{{y_{{\left( {face} \right)}} }} \times \left( {c + t} \right) \times \frac{b \times t}{L} $$

#### Face sheet wrinkling

Face wrinkling is a local elastic instability of the face sheet characterized by short-wavelength elastic buckling. This phenomenon only occurs in a compressive face sheet that is supported by a unilateral elastic foundation, such as foam. The critical wrinkling force, denoted as ${P}_{{wr}_{(face)}}$, can be determined by the following calculation^44^:

43 $$ P_{{wr_{{\left( {face} \right)}} }} = 4 \times \sigma_{{wr_{{\left( {face} \right)}} }} \times \left( {c + t} \right) \times \frac{b \times t}{L} $$

Consequently, the stress that leads to wrinkling in the face sheet, denoted to as $\sigma_{{wr_{{\left( {face} \right)}} }}$, is calculated by^45^:

44 $$ \sigma_{{wr_{{\left( {face} \right)}} }} = \frac{{\pi^{2} }}{12} \times \left( {\frac{t}{{l_{f} }}} \right)^{2} \times \frac{{E_{s} }}{{\left( {1 - \nu^{2} } \right)}} $$

where ${l}_{f}$ is the distance between the attachment area of core cells to the face-sheet (Fig. 3b).

#### Core shear

In sandwich beam the transverse shear force is carried by the core. The collapse force of the core shear failure is estimated as^43^:

45 $$ P_{{s_{{\left( {core} \right)}} }} = \left[ {2 \times \sigma_{{y_{{\left( {face} \right)}} }} \times \frac{{b \times t^{2} }}{L}} \right] + \left[ {2 \times \tau_{{y_{{\left( {core} \right)}} }} \times \frac{b \times c}{L} \times \left( {L + 2h} \right)} \right] $$

where $h$ is the distance from the support to the unrestrained end of the beam, and the shear yield strength for the core is presented as ${\tau }_{{y}_{(core)}}$.

#### Indentation

In sandwich beam, the failure due to indentation involves the development of four plastic hinges within the upper face sheet near the indenter, along with the compressive collapse of the underlying core. Consequently, the indentation’s collapse load is determined by^43,44^:

46 $$ P_{IND} = \left[ {b \times t \times \sqrt {\sigma_{{y_{{\left( {core} \right)}} }} \times \sigma_{{y_{{\left( {face} \right)}} }} } } \right] + \sigma_{{y_{{\left( {core} \right)}} }} \times d_{1} \times b $$

By regarding, ${d}_{1}$ is the width of the loading head, when employing cylindrical loading heads and supports, Eq. (46) should be substituted with^45,46^:

47 $$ P_{IND} = b \times t \times \sqrt {\sigma_{{y_{{\left( {core} \right)}} }} \times \sigma_{{y_{{\left( {face} \right)}} }} } $$

However, the critical load predictions of face yield mode and indentation mode are significantly influenced by the properties of both the face sheet and core material.

### Failure mode maps

The failure mode maps for sandwich beams under three-point bending by employing dimensionless parameters $t/L$ and $c/L$, can be generated using Eqs. (43), (45), and (47). This map is divided into three distinct regions, each dominated by a primary failure mechanism. Also, the transition lines are governed as the following equations:

48 $$ \frac{c}{L} = \left[ {\frac{1}{2} \times \sqrt {\frac{{\sigma_{{y_{{\left( {core} \right)}} }} }}{{\sigma_{{y_{{\left( {face} \right)}} }} }}} } \right] - \frac{t}{L} $$

49 $$ \frac{c}{L} = \left[ {\left( {1 + 2 \times \frac{h}{L}} \right) \times \frac{{\tau_{{y_{{\left( {core} \right)}} }} }}{{\sigma_{{y_{{\left( {face} \right)}} }} }} - 2 \times \frac{t}{L}} \right]^{ - 1} \times \left[ \frac{t}{L} \right]^{2} $$

50 $$ \frac{c}{L} = \left[ {\frac{t}{L} \times \sqrt {\frac{{\sigma_{{y_{{\left( {core} \right)}} }} }}{{\sigma_{{y_{{\left( {face} \right)}} }} }}} - \left( \frac{t}{L} \right)^{2} } \right] \times \left[ {\left( {1 + 2 \times \frac{h}{L}} \right) \times \frac{{\tau_{{y_{{\left( {core} \right)}} }} }}{{\sigma_{{y_{{\left( {face} \right)}} }} }}} \right]^{ - 1} $$

These transition lines depend on the strength materials of face-sheet and core and the values of ${\tau }_{{y}_{(core)}}$, and ${\sigma }_{{y}_{(core)}}$ are estimated by Eqs. (26) and (33), respectively.

### Ethics approval and consent to participate

The authors provided informed consent to enrolment in this study.

## Results and discussion

### Analytical models

**Figure 13:**
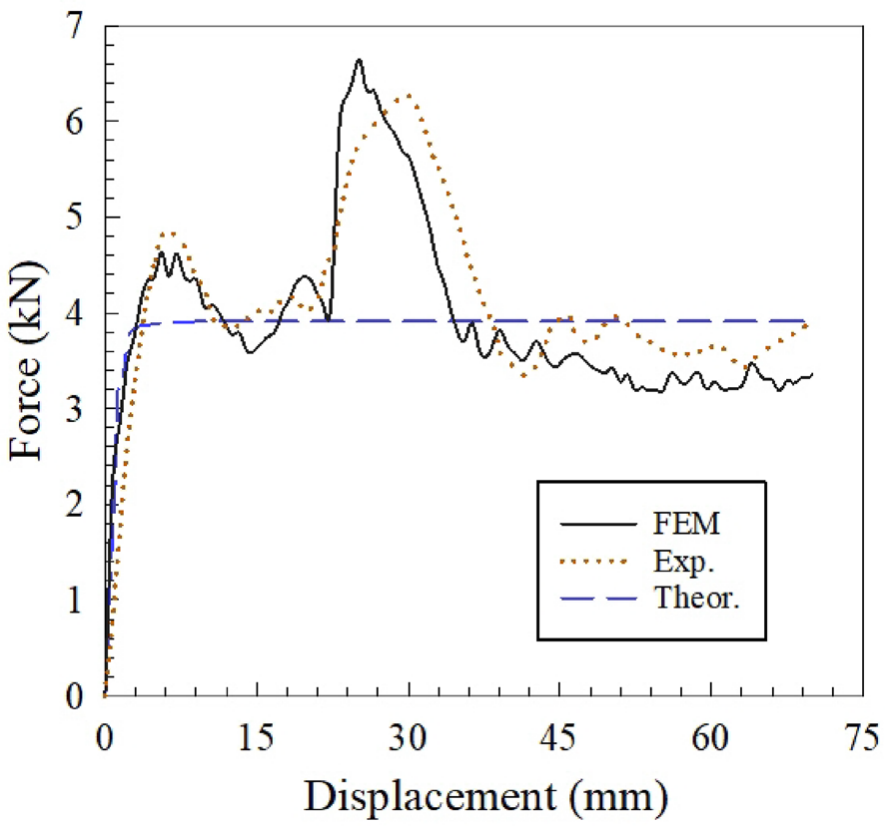
Comparison Experimental, Numerical, and Analytical Results of C332^15^.

Initially, employing the presented parameters in the numerical investigation^15^, the analytical equations of the previous sections have been evaluated. Hence, the comparison of the experimental, numerical and analytical output results of sample C332 is shown in Fig. 13, and a detailed breakdown of experimental and numerical sample C332 has been provided in the supplementary section.

Also, their considered parameters are presented in Table 2. The results showed that cell No.3 has had a minimal deformation, calculated 8.8 percent, whereas cell number 4 collapsed by a significant value, 39.5 percent. Moreover, the mean force and energy absorption of additional samples subjected to a 70 mm deformation have been calculated, and the comparison of the experimental and analytical results is delineated in Table 3. It is an acceptable congruence has been found between the experimental and analytical results, such that the maximum discrepancy approached approximately 10 percent. (More details in Appendix II).

**Table 2:** Considered parameters for estimating the mean force, *P* load, for sample C332.

Sample	Parameters	
	Unit in millimeter	Dimension less
*C332*	$L=150$, $b=80$, $h=50$, $d\cong 46.8$ , $t=2$ , ${L}_{1}\cong 66.7$, ${L}_{2}\cong 22$, $w=8$, $a=3$, ${N}_{c}=18$,$m=2$	${\beta }_{1}, \dots , {\beta }_{6}=1$ ${\beta }_{7}, \dots , {\beta }_{12}\cong 0.088$ ${\beta }_{13}, \dots , {\beta }_{18}\cong 0.395$

**Table 3:** Comparison of experimental and analytical results.

Nos	Sample	*MCF* (kN)—analytical	*EA* (J)		Error
			Experimental^15^	Analytical	
1	C132*	2.408	160.795	168.533	% 4.81
2	*C161*	2.776	205.830	194.347	% 5.58
3	*C163*	4.818	310.711	337.252	% 8.54
4	*C192*	4.460	283.573	312.204	% 10.1
5	*C231*	0.930	64.1540	65.1070	% 1.49
6	*C233*	2.315	170.333	162.075	% 4.85
7	*C262*	2.083	160.464	145.794	% 9.14
8	*C291*	1.745	112.742	122.150	% 8.34
9	*C293*	3.532	237.802	247.256	% 3.98
10	*C332*	3.913	290.782	273.901	% 5.81
11	*C361*	5.617	405.345	393.186	% 3.00
12	*C363*	8.171	592.529	571.966	% 3.47
13	*C392*	9.324	720.620	652.661	% 9.43

*Define the sample as follows: CXYZ, X: the type of core size, Y: the number of rows, Z: thickness of face-sheet.

### The failure maps

**Figure 14:**
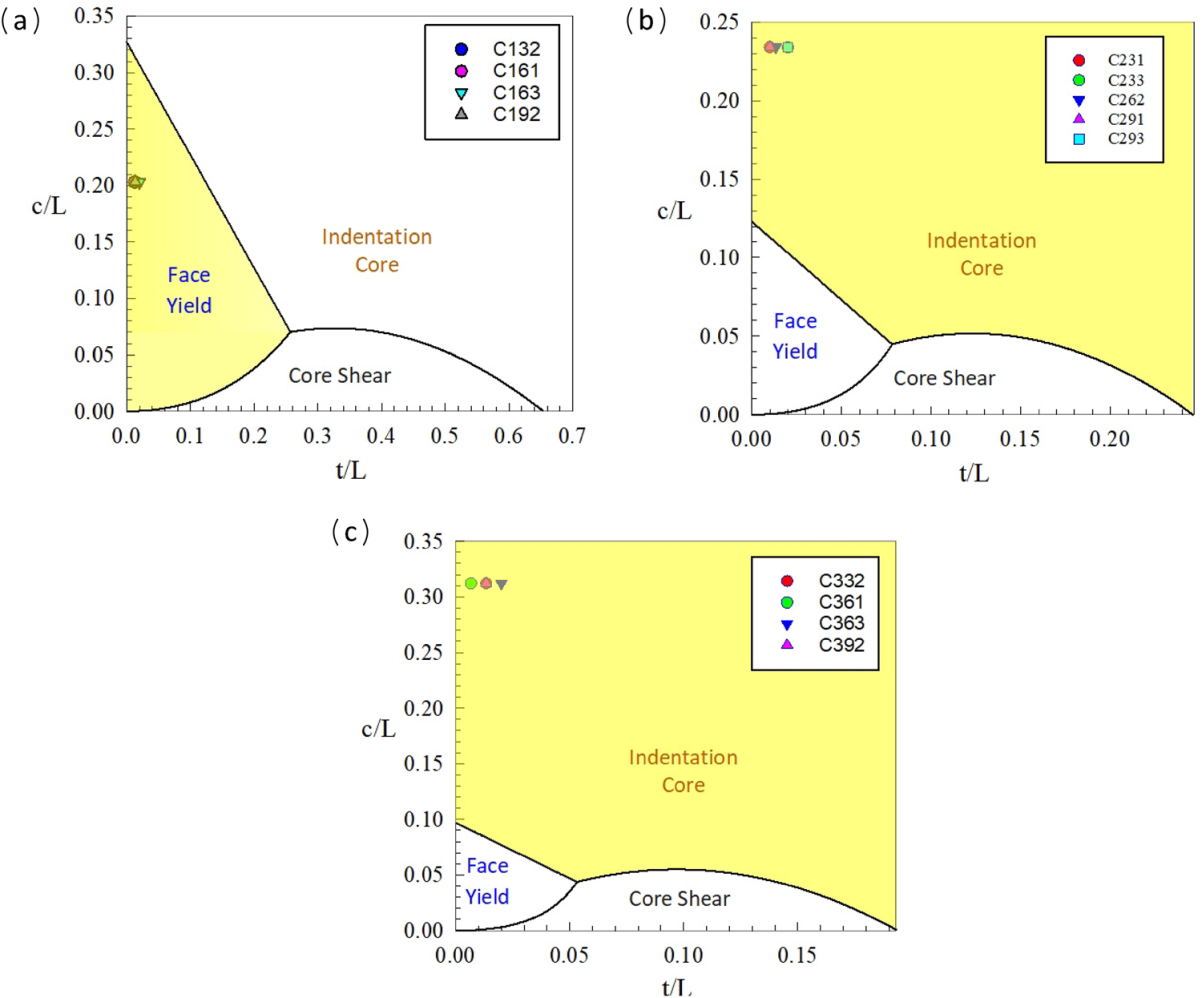
Failure mode maps of the core size; (**a**) Type 1, (**b**) Type 2, (**c**) Type 3.

In consideration equations of the failure maps, the transition lines are extremely deponed on the strength of core, such that the failure mode of the sandwich panels under the bending load is affected by the effective parameters of the compressive strength and the shear strength of core. In Fig. 14, the failure maps of three different core size types are depicted. In these figures, the failure maps categorized into three distinct domains: face yield, indentation core, and core shear. The obtained outcomes conspicuously show that the failure type of the sandwich panels with the core size type 1 be associated with the face yield region, while for both core types 2 and 3, the failure occurs within the indentation domain. As a result of Fig. 14, when the face-sheets are thicker and stronger, the failure mode tends to shift towards the core shear domain.

**Figure 15:**
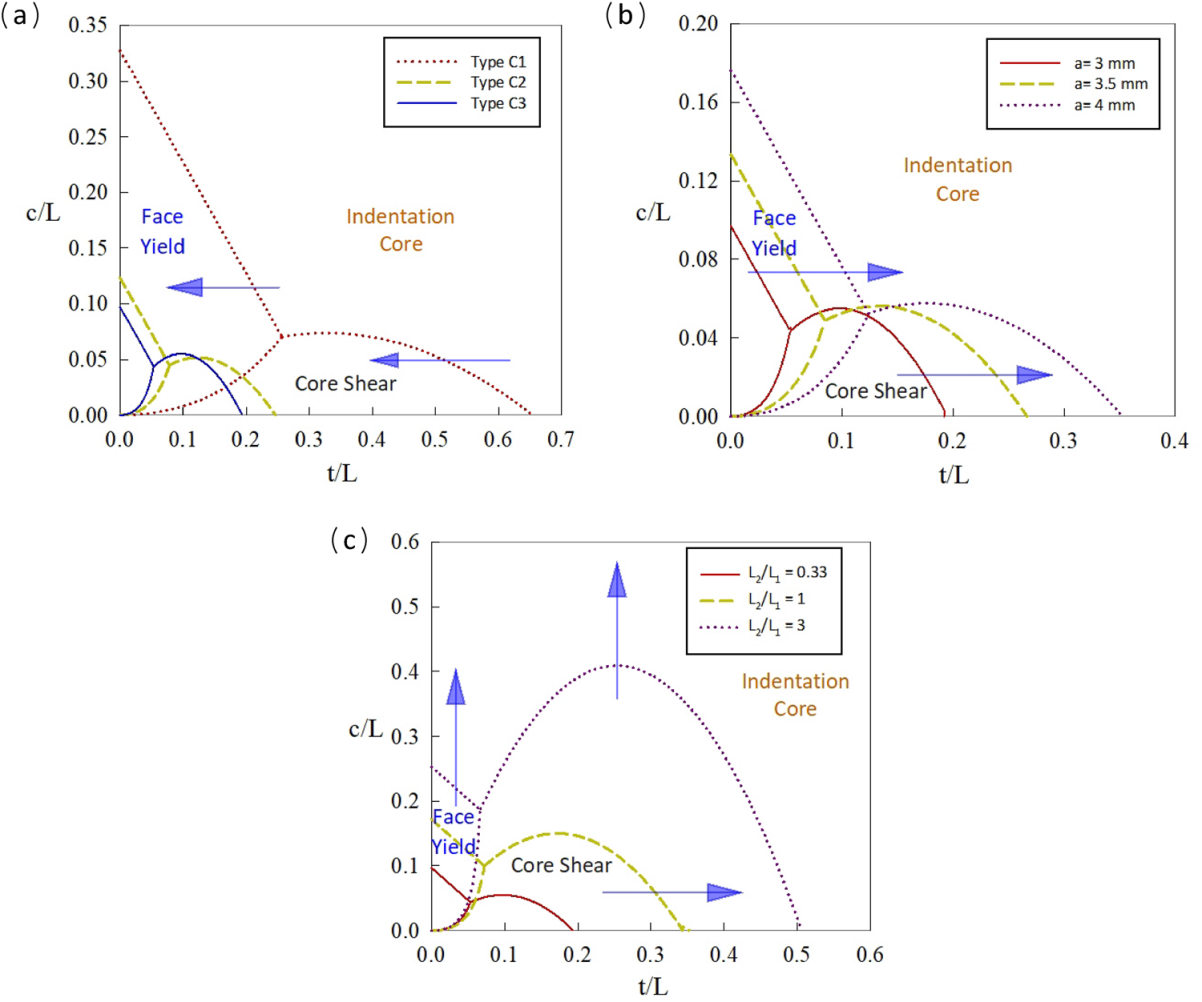
Comparison of failure mode maps by increasing; (**a**) core type size, (**b**) strand thickness of core, (**c**) ratio of Length ${L}_{2}$ to ${L}_{1}$.

The results to variations in the core size, as illustrated in Fig. 15a, demonstrate that an increase in the core size, from Type C1 to Type C3, leads to a reduction of both the face yield and core shear regions within the failure map. The thickness of the core cell stands is another parameter which significantly effect on the compressive and shear strengths of the core of a sandwich panel beam. As presented in Fig. 15b, with the increasing thickness, the transition lines caused to the right side, and the extension of the failure modes of face yield and core shear domain is observed. Moreover, another crucial parameter that can affect the failure of shape modes in sandwich panel beams is related to the ratio of ${L}_{2}$ to ${L}_{1}$. As this ratio increases, the lines associated with the core shear region move upwards, and it shows that the failure mode can be controlled by keeping other parameters constant and increasing this ratio (Fig. 15c).

## Conclusion

An analytical study on the failure mechanisms of the sandwich panel beams with steel face sheets and lattice core under quasi-static three-point bending was conducted. Hence, efforts were exerted to develop an analytical relationship for the calculation of mean force and energy absorption, employing the principles of elastic and plastic deformation governing beams. Four main failure modes have been recognized in the context of sandwich panel beams; face yield, wrinkling of the face-sheet due to compression, core shear, and indentation core failure. These equations demonstrate that the critical load predictions for the failure mode from the output models are strongly influenced by the material properties of the sheets. The theoretical predictions concerning the failure mode, mean force, and energy absorption align closely with the experimental and numerical results. The results obtained clearly indicate that the failure mode type observed in sandwich panels with core size type 1 is predominantly associated with the face yield region. Meanwhile, for core types 2 and 3, failure predominantly occurs within the indentation core domain. Also, the final results are demonstrated that the failure face yield and core shear regions are significantly influenced by variations in core sizes. The thickness of the core cell strands was also found to crucially affect the compressive and shear strengths of sandwich panel beams. Furthermore, the ratio of ${L}_{2}$ to ${L}_{1}$ was identified as a critical factor in the control of failure modes by adjusting this ratio and keeping other parameters constant. Hence, it is necessary to evaluate the collapse mechanism in the design of sandwich beams in addition to their stiffness.

### Supplementary Information

Supplementary Information.

## Data Availability

The data supporting the outcomes of this study are available based on the request from the corresponding author.
