# Supplementary material for: Analysis of deformation and failure mechanism of sandwich beams with lattice core under three-point bending load
Source: Sci Rep. 2024 Jun 10;14:13302. doi: 10.1038/s41598-024-64198-y (PMC11165008; doi:10.1038/s41598-024-64198-y)
Supplement: Supplementary file 1 — Supplementary Information. [file 41598_2024_64198_MOESM1_ESM.docx]

Appendix I

| Notation and nomenclature | |
| --- | --- |
| **Symbols** | **Definition** |
| $E$ | Elastic modulus |
| $E_{s}$ | Face sheet young’s modulus and the material of core |
| $E_{c}$ | Core young’s modulus |
| $E_{T}$ | Total plastic strain energy |
| $E_{face}$ | Plastic strain energy of face sheet |
| $E_{core}$ | Plastic strain energy of core |
| $I$ | The moment of inertia about the neutral axis |
| ${(EI)}_{eq}$ | Equivalent flexural stiffness |
| $G$ | Shear modulus |
| $G_{c}$ | Maximum shear modulus of a core cell |
| $A$ | Cross section area |
| $A_{c}$ | Cross section area of a cell on the $xz$-plane |
| $V_{T}$ | Volume of unit cells |
| $V_{C}$ | Volume of cell members |
| ${(AG)}_{eq}$ | Equivalent shear stiffness |
| $\rho$ | Density |
| $\bar{\rho}$ | Core density of sandwich panels |
| $\nu$ | Poisson's ratio |
| $\sigma_{y}$ | Yield stress |
| $\sigma_{u}$ | Ultimate stress |
| $\sigma_{y_{(face)}}$ | Yield strength of the face-sheet |
| ${\sigma_{y}}_{(core)}$ | Maximum compressive stress for the core |
| $\tau_{yx}$ | Shear stress on the $xy$-plane |
| ${\tau_{y}}_{(core)}$ | Maximum shear stress for the core |
| $f_{N}$ | Tensile force on a beam |
| $f_{x}$ | Force in the $x$-direction |
| $f_{y}$ | Force in the $y$-direction |
| $f_{x_{local}}$ | Force in the $x_{local}$-direction |
| $f_{y_{local}}$ | Force in the $y_{local}$-direction |
| $P$ | Bending load |
| ${P_{cr}}_{(core)}$ | Maximum buckling load acting upon a cell |
| ${P_{y}}_{(face)}$ | Face-sheet yielding load |
| ${P_{wr}}_{(face)}$ | Face-sheet wrinkling load |
| ${P_{s}}_{(core)}$ | The core's shear load |
| $P_{IND}$ | Indentation collapse load |
| $\delta$ | Sandwich beam deflection (the sum of flexural and shear deflections) |
| $\delta_{x}$ | Deflection in the $x$-direction |
| $U_{yb}$ | The displacement magnitude at the center of the beam (in the $y$-direction) |
| $U_{yc}$ | The displacement magnitude in each cell (in the $y$-direction) |
| $y_{c}$ | Distance from the neutral axis |
| $l_{f}$ | The effective wrinkling length |
| $\lambda$ | Distance from the plastic hinge on the face-sheet to the applied force point |
| $M$ | Bending moment on a beam |
| $M_{A}$ | Bending moment about point A |
| $M_{B}$ | Bending moment about point B |
| $M_{p}$ | Bending moment about plastic hinge point |
| $\left( M_{p} \right)_{face}$ | Plastic moment of face sheets |
| $\left( M_{p} \right)_{cell}$ | Plastic moment of the cell joints |
| $a$ | Strand thickness |
| $d_{1}$ | The loading head width |
| $b$ | Panel width |
| $c$ | Core height or core thickness |
| $d$ | Panel height |
| $h$ | Overhang distance beyond the support |
| $L$ | The span between the outer supports |
| $L_{1}$ | Long way of the opening, the clear opening of the diamond in the long direction of the diamond (or LWO) |
| $L_{2}$ | Short way of the opening, the clear opening of the diamond across the short direction of the diamond (or SWO) |
| $L_{s}$ | Strand length |
| $2s$ | Spacing between of points where adjacent strands meet (bond) |
| $w$ | Strand width |
| $t$ | Thickness of upper or lower face sheet in each panel |
| $N_{c}$ | The quantity of total cells of a core |
| $N_{j}$ | The quantity of total joints of core |
| $m$ | The quantity of fully deformed central cells |
| $\omega$ | Angular velocity |
| $EA$ | Energy absorption capacity of the absorber |
| $MCF$ | Mean Crush Force |

Appendix II

Supplementary Figures and Tables:

|  | 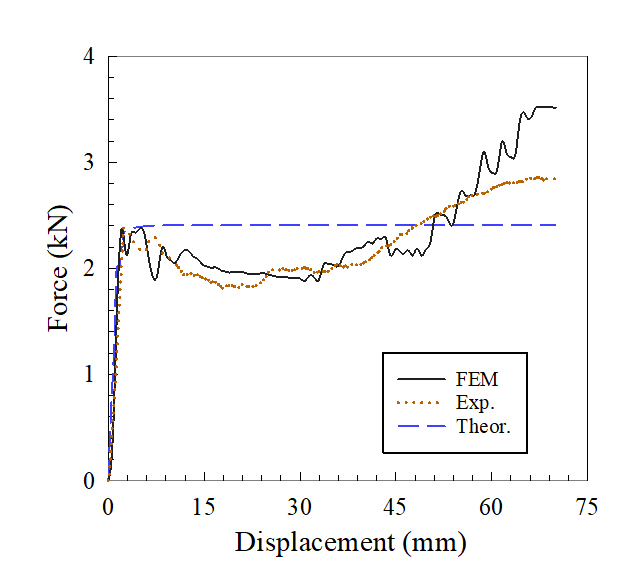 |  | 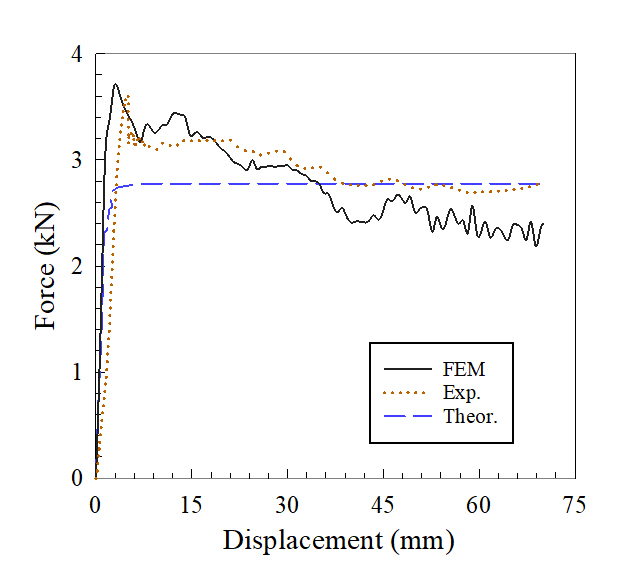 |
| --- | --- | --- | --- |
|  | 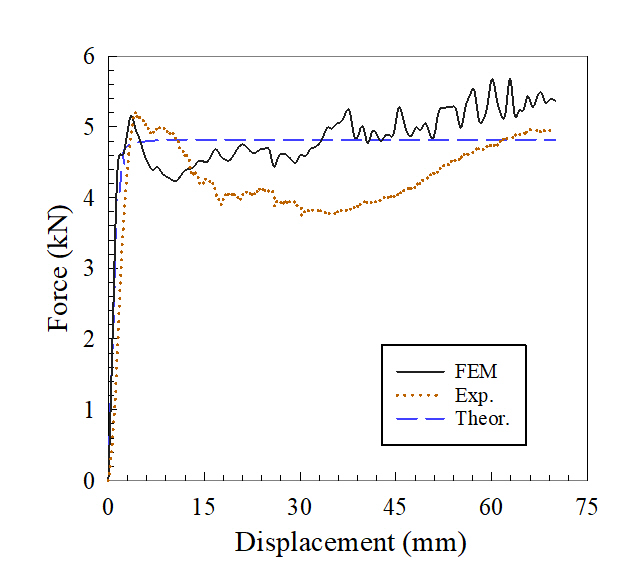 |  | 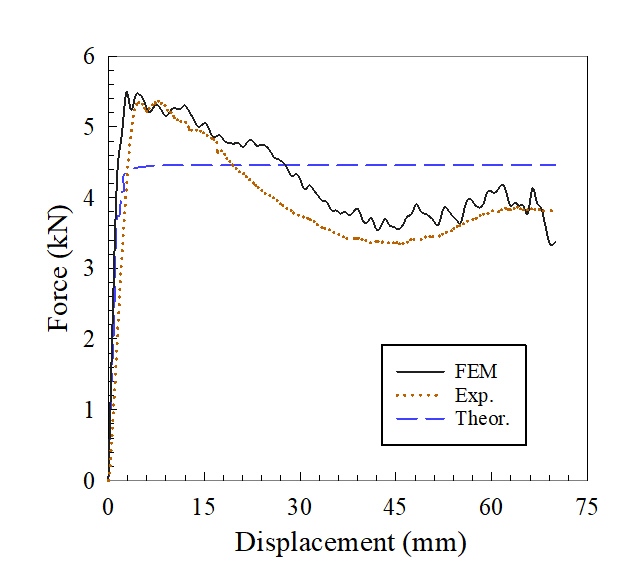 |
| Comparison Experimental, Numerical [14], and Analytical Results of; (a)C132, (b) C161, (c) C163, (d) C192 | | | |

|  | 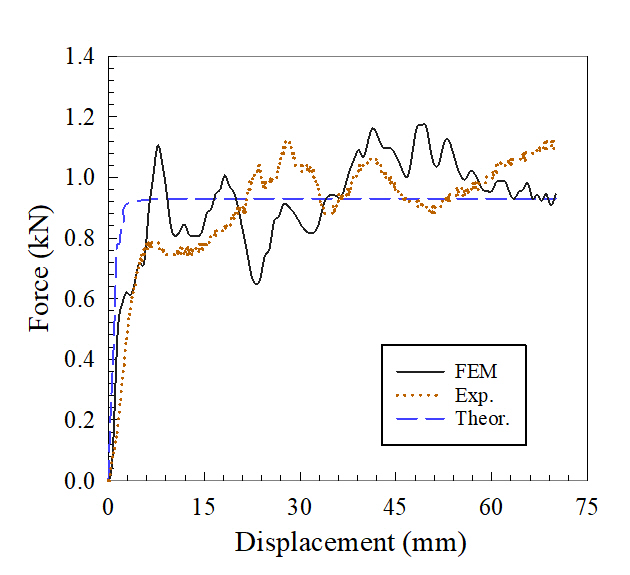 | |  | 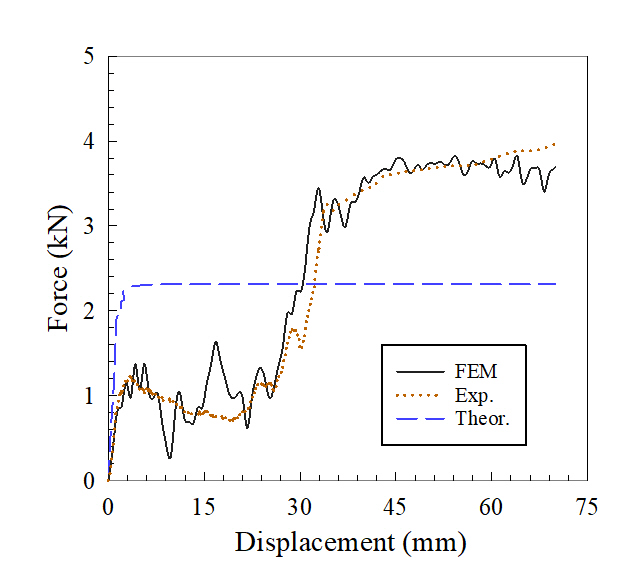 |
| --- | --- | --- | --- | --- |
|  | 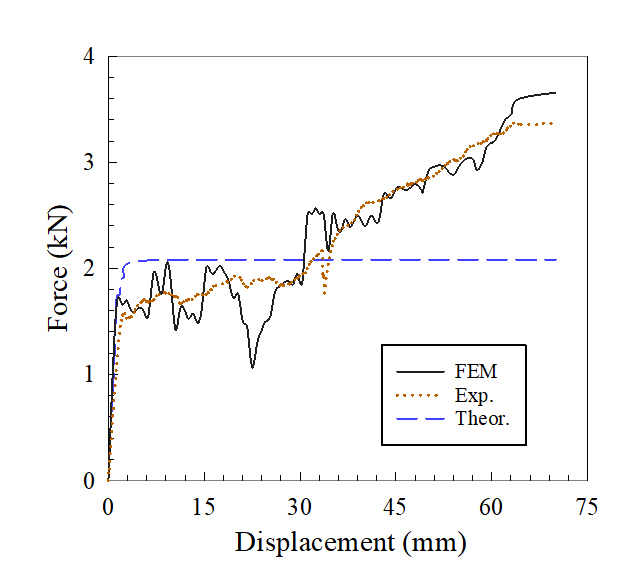 | |  | 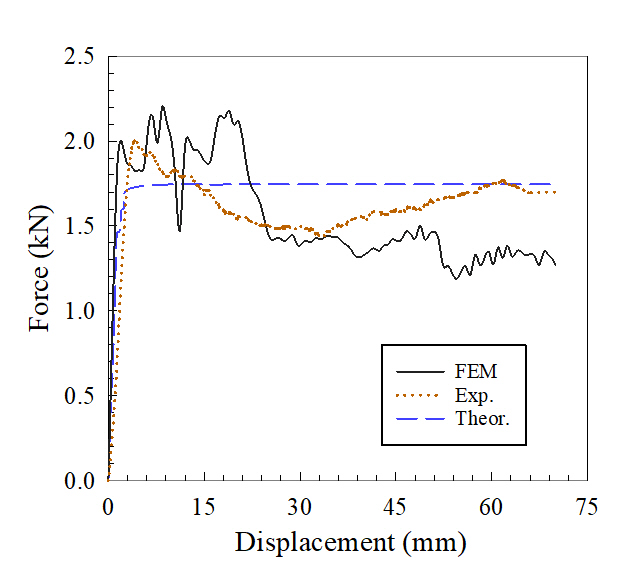 |
|  | | 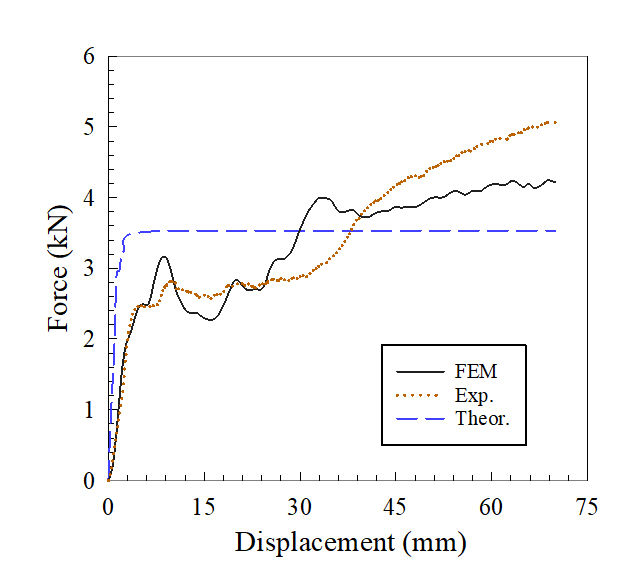 | | |
| Comparison Experimental, Numerical [14], and Analytical Results of; (a)C231, (b) C233, (c) C262, (d) C291 and (f) C293 | | | | |

|  | 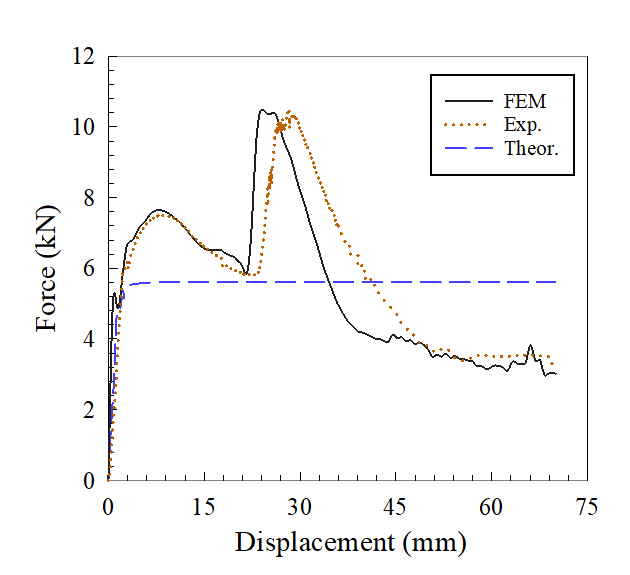 | |  | 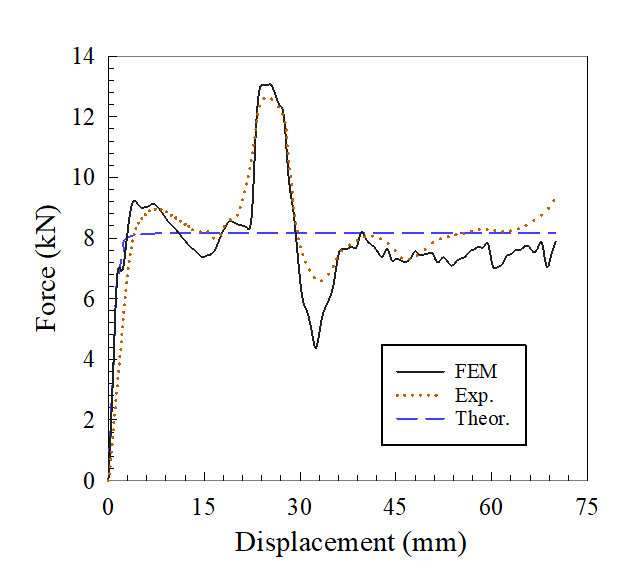 |
| --- | --- | --- | --- | --- |
|  | | 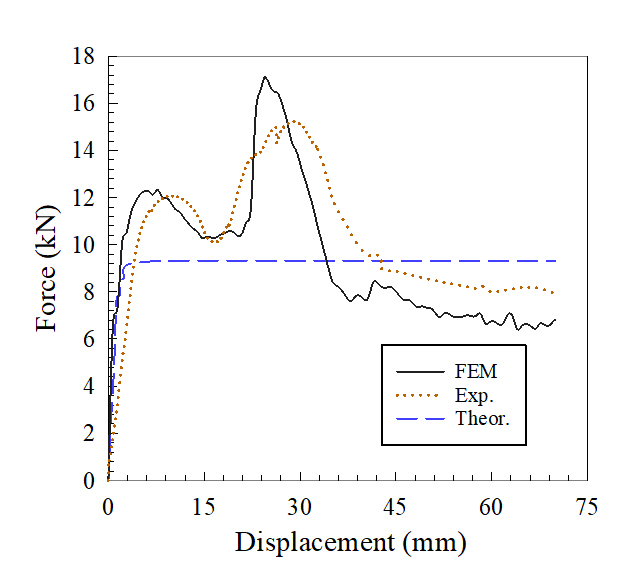 | | |
| Comparison Experimental, Numerical [14], and Analytical Results of; (a)C361, (b) C363 and (c) C392 | | | | |

| 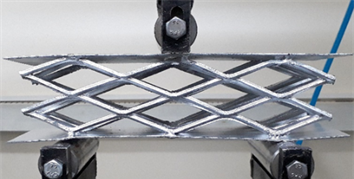 | 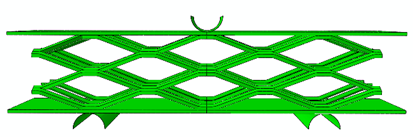 |
| --- | --- |
| 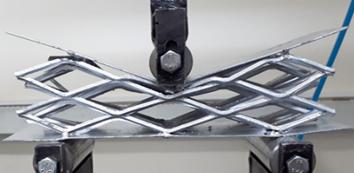 | 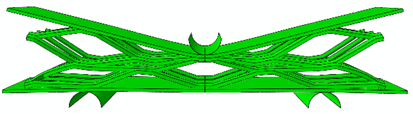 |
| 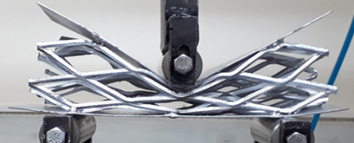 | 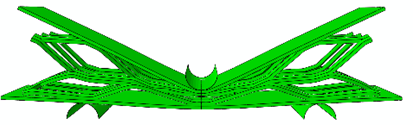 |
| 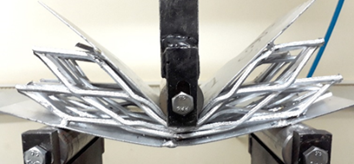 | 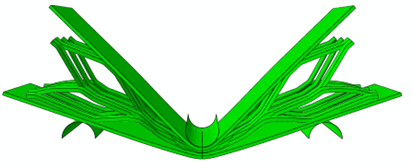 |
| Comparison Experimental and Numerical deformation of C332 [14] | |

| Considered Parameters for calculating *P* load for other samples | | | | |
| --- | --- | --- | --- | --- |
| **No.** | **Sample** | **Parameters** | | |
|  |  | *Unit in millimeter* | *Dimension less* | |
|  | *C132* | $L=150$, $b=80$ , $h=50$,  $d\cong34.5$ , $t=2$ , $L_{1}\cong16.7$, $L_{2}\cong7$,  $w=3$, $a=1$, $N_{c}=144$, $m=5$ | $\beta_{1}, \ldots, \beta_{30}=1$ | $\beta_{31}, \ldots, \beta_{36}\cong0.32$ |
|  |  |  | $\beta_{37}, \ldots, \beta_{42}\cong0.22$ | $\beta_{43}, \ldots, \beta_{48}\cong0.11$ |
|  |  |  | $\beta_{49}, , \beta_{54}\cong0.13$ | $\beta_{55}, \ldots, \beta_{60}\cong0.24$ |
|  |  |  | $\beta_{61}, \ldots, \beta_{66}\cong0.17$ | $\beta_{67}, \ldots, \beta_{72}\cong0.17$ |
|  |  |  | $\beta_{73}, \ldots, \beta_{78}\cong0.20$ | $\beta_{79}, \ldots, \beta_{84}\cong0.38$ |
|  |  |  | $\beta_{85}, \ldots, \beta_{90}\cong0.57$ | $\beta_{91}, \ldots, \beta_{96}\cong0.04$ |
|  |  |  | $\beta_{97}, \ldots, \beta_{102}\cong0.21$ | $\beta_{103}, \ldots, \beta_{108}\cong0.37$ |
|  |  |  | $\beta_{109}, \ldots, \beta_{114}\cong0.61$ | $\beta_{115}, \ldots, \beta_{120}\cong0.52$ |
|  |  |  | $\beta_{121}, \ldots, \beta_{126}\cong0.72$ | $\beta_{127}, \ldots, \beta_{132}\cong0.94$ |
|  |  |  | $\beta_{133}, \ldots, \beta_{138}\cong0.90$ | $\beta_{139}, \ldots, \beta_{144}\cong0$ |
|  | *C161* | $L=150$, $b=80$ , $h=50$,  $d\cong34.1$ , $t=1.8$ , $L_{1}\cong16.7$, $L_{2}\cong7$,  $w=3$, $a=1$, $N_{c}=288$, $m=6$ | $\beta_{1}, \ldots, \beta_{72}=1$ | $\beta_{73}, \ldots, \beta_{84}\cong0.19$ |
|  |  |  | $\beta_{85}, \ldots, \beta_{96}\cong0.20$ | $\beta_{97}, \ldots, \beta_{108}\cong0.11$ |
|  |  |  | $\beta_{109}, \ldots, \beta_{120}\cong0.04$ | $\beta_{121}, \ldots, \beta_{132}\cong0.08$ |
|  |  |  | $\beta_{133}, \ldots, \beta_{144}\cong0.58$ | $\beta_{145}, \ldots, \beta_{156}\cong0.13$ |
|  |  |  | $\beta_{157}, \ldots, \beta_{168}\cong0.12$ | $\beta_{169}, \ldots, \beta_{180}\cong0.10$ |
|  |  |  | $\beta_{181}, \ldots, \beta_{192}\cong0.12$ | $\beta_{193}, \ldots, \beta_{204}\cong0.29$ |
|  |  |  | $\beta_{205}, \ldots, \beta_{216}\cong0.02$ | $\beta_{217}, \ldots, \beta_{228}\cong0.08$ |
|  |  |  | $\beta_{229}, \ldots, \beta_{240}\cong0.18$ | $\beta_{241}, \ldots, \beta_{252}\cong0.25$ |
|  |  |  | $\beta_{253}, \ldots, \beta_{264}\cong0.16$ | $\beta_{265}, \ldots, \beta_{276}\cong0.55$ |
|  |  |  | $\beta_{277}, \ldots, \beta_{288}\cong0$ |  |

| Considered Parameters for calculating *P* load for other samples | | | | |
| --- | --- | --- | --- | --- |
| \| **No.** \| **Sample** \| **Parameters** \| \| \| --- \| --- \| --- \| --- \| \| *Unit in millimeter* \| *Dimension less* \| | | | | |
|  | *C163* | $L=150$, $b=80$ , $h=50$,  $d\cong36.5$ , $t=3$ , $L_{1}\cong16.7$, $L_{2}\cong7$,  $w=3$, $a=1$, $N_{c}=288$, $m=6$ | $\beta_{1}, \ldots, \beta_{72}=1$ | $\beta_{73}, \ldots, \beta_{84}\cong0.62$ |
|  |  |  | $\beta_{85}, \ldots, \beta_{96}\cong0.50$ | $\beta_{97}, \ldots, \beta_{108}\cong0.33$ |
|  |  |  | $\beta_{109}, \ldots, \beta_{120}\cong0.21$ | $\beta_{121}, \ldots, \beta_{132}\cong0.24$ |
|  |  |  | $\beta_{133}, \ldots, \beta_{144}\cong0.33$ | $\beta_{145}, \ldots, \beta_{156}\cong0.28$ |
|  |  |  | $\beta_{157}, \ldots, \beta_{168}\cong0.31$ | $\beta_{169}, \ldots, \beta_{180}\cong0.51$ |
|  |  |  | $\beta_{181}, \ldots, \beta_{192}\cong0.67$ | $\beta_{193}, \ldots, \beta_{204}\cong0.10$ |
|  |  |  | $\beta_{205}, \ldots, \beta_{216}\cong0.26$ | $\beta_{217}, \ldots, \beta_{228}\cong0.45$ |
|  |  |  | $\beta_{229}, \ldots, \beta_{240}\cong0.62$ | $\beta_{241}, \ldots, \beta_{252}\cong0.48$ |
|  |  |  | $\beta_{253}, \ldots, \beta_{264}\cong0.76$ | $\beta_{265}, \ldots, \beta_{276}\cong0.79$ |
|  |  |  | $\beta_{277}, \ldots, \beta_{288}\cong0$ |  |
|  | *C192* | $L=150$, $b=80$ , $h=50$,  $d\cong34.5$ , $t=2$ , $L_{1}\cong16.7$, $L_{2}\cong7$,  $w=3$, $a=1$, $N_{c}=432$, $m=5$ | $\beta_{1}, \ldots, \beta_{90}=1$ | $\beta_{91}, \ldots, \beta_{108}\cong0.18$ |
|  |  |  | $\beta_{109}, \ldots, \beta_{126}\cong0.24$ | $\beta_{127}, \ldots, \beta_{144}\cong0.22$ |
|  |  |  | $\beta_{145}, \ldots, \beta_{162}\cong0.07$ | $\beta_{163}, \ldots, \beta_{180}\cong0.10$ |
|  |  |  | $\beta_{181}, \ldots, \beta_{198}\cong0.14$ | $\beta_{199}, \ldots, \beta_{216}\cong0.16$ |
|  |  |  | $\beta_{217}, \ldots, \beta_{234}\cong0.08$ | $\beta_{235}, \ldots, \beta_{252}\cong0.18$ |
|  |  |  | $\beta_{253}, \ldots, \beta_{270}\cong0.40$ | $\beta_{271}, \ldots, \beta_{288}\cong0.05$ |
|  |  |  | $\beta_{289}, \ldots, \beta_{306}\cong0.02$ | $\beta_{307}, \ldots, \beta_{324}\cong0.16$ |
|  |  |  | $\beta_{325}, \ldots, \beta_{342}\cong0.19$ | $\beta_{343}, \ldots, \beta_{360}\cong0.03$ |
|  |  |  | $\beta_{361}, \ldots, \beta_{378}\cong0.22$ | $\beta_{379}, \ldots, \beta_{396}\cong0.52$ |
|  |  |  | $\beta_{397}, \ldots, \beta_{414}\cong0.73$ | $\beta_{415}, \ldots, \beta_{432}\cong0$ |

| \| Considered Parameters for calculating *P* load for other samples \| \| --- \| \| \| **No.** \| **Sample** \| **Parameters** \| \| \| --- \| --- \| --- \| --- \| \| *Unit in millimeter* \| *Dimension less* \| \| | | | | |
| --- | --- | --- | --- | --- | --- | --- | --- | --- | --- | --- | --- | --- |
|  | *C231* | $L=150$, $b=80$ , $h=50$,  $d\cong38.1$ , $t=1.5$ , $L_{1}\cong34.7$, $L_{2}\cong11$,  $w=4$, $a=0.75$, $N_{c}=54$, $m=3$ | $\beta_{1}, \ldots, \beta_{18}=1$ | $\beta_{19}, \ldots, \beta_{24}\cong0.09$ |
|  |  |  | $\beta_{25}, \ldots, \beta_{30}\cong0.70$ | $\beta_{31}, \ldots, \beta_{36}\cong0.63$ |
|  |  |  | $\beta_{37}, \ldots, \beta_{42}\cong0.49$ | $\beta_{43}, \ldots, \beta_{48}\cong0.64$ |
|  |  |  | $\beta_{49}, \ldots, \beta_{54}\cong0.59$ |  |
|  | *C233* | $L=150$, $b=80$ , $h=50$,  $d\cong41.1$ , $t=3$ , $L_{1}\cong34.7$, $L_{2}\cong11$,  $w=4$, $a=0.75$, $N_{c}=54$, $m=3$ | $\beta_{1}, \ldots, \beta_{18}=1$ | $\beta_{19}, \ldots, \beta_{24}\cong0.77$ |
|  |  |  | $\beta_{25}, \ldots, \beta_{30}\cong0.87$ | $\beta_{31}, \ldots, \beta_{36}\cong0.85$ |
|  |  |  | $\beta_{37}, \ldots, \beta_{42}\cong0.88$ | $\beta_{43}, \ldots, \beta_{48}\cong0.92$ |
|  |  |  | $\beta_{49}, \ldots, \beta_{54}\cong0.95$ |  |
|  | *C262* | $L=150$, $b=80$ , $h=50$,  $d\cong39.1$ , $t=2$ , $L_{1}\cong34.7$, $L_{2}\cong11$,  $w=4$, $a=0.75$, $N_{c}=108$, $m=2$ | $\beta_{1}, \ldots, \beta_{24}=1$ | $\beta_{25}, \ldots, \beta_{36}\cong0.56$ |
|  |  |  | $\beta_{37}, \ldots, \beta_{48}\cong0.20$ | $\beta_{49}, \ldots, \beta_{60}\cong0.70$ |
|  |  |  | $\beta_{61}, \ldots, \beta_{72}\cong0.20$ | $\beta_{73}, \ldots, \beta_{84}\cong0.86$ |
|  |  |  | $\beta_{85}, \ldots, \beta_{96}\cong0.17$ | $\beta_{97}, \ldots, \beta_{108}\cong0.60$ |
|  | *C291* | $L=150$, $b=80$ , $h=50$,  $d\cong38.1$ , $t=1.5$ , $L_{1}\cong34.7$, $L_{2}\cong11$,  $w=4$, $a=0.75$, $N_{c}=162$, $m=3$ | $\beta_{1}, \ldots, \beta_{54}=1$ | $\beta_{55}, \ldots, \beta_{72}\cong0.68$ |
|  |  |  | $\beta_{73}, \ldots, \beta_{90}\cong0.38$ | $\beta_{91}, \ldots, \beta_{108}\cong0.31$ |
|  |  |  | $\beta_{109}, \ldots, \beta_{126}\cong0.23$ | $\beta_{127}, \ldots, \beta_{144}\cong0.57$ |
|  |  |  | $\beta_{145}, \ldots, \beta_{162}\cong0$ |  |
|  | *C293* | $L=150$, $b=80$ , $h=50$,  $d\cong41.1$ , $t=3$ , $L_{1}\cong34.7$, $L_{2}\cong11$,  $w=4$, $a=0.75$, $N_{c}=162$, $m=3$ | $\beta_{1}, \ldots, \beta_{54}=1$ | $\beta_{55}, \ldots, \beta_{72}\cong0.54$ |
|  |  |  | $\beta_{73}, \ldots, \beta_{90}\cong0.71$ | $\beta_{91}, \ldots, \beta_{108}\cong0.64$ |
|  |  |  | $\beta_{109}, \ldots, \beta_{126}\cong0.84$ | $\beta_{127}, \ldots, \beta_{144}\cong0.86$ |
|  |  |  | $\beta_{145}, \ldots, \beta_{162}\cong86$ |  |
|  | *C361* | $L=150$, $b=80$ , $h=50$,  $d\cong48.8$ , $t=1$ , $L_{1}\cong66.7$, $L_{2}\cong22$,  $w=8$, $a=3$, $N_{c}=36$, $m=2$ | $\beta_{1}, \ldots, \beta_{12}=1$ | $\beta_{13}, \ldots, \beta_{24}\cong0.26$ |
|  |  |  | $\beta_{25}, \ldots, \beta_{36}\cong0$ |  |
|  | *C363* | $L=150$, $b=80$ , $h=50$,  $d\cong52.8$ , $t=3$ , $L_{1}\cong66.7$, $L_{2}\cong22$,  $w=8$, $a=3$, $N_{c}=36$, $m=2$ | $\beta_{1}, \ldots, \beta_{12}=1$ | $\beta_{13}, \ldots, \beta_{24}\cong0.13$ |
|  |  |  | $\beta_{25}, \ldots, \beta_{36}\cong0.39$ |  |
|  | *C392* | $L=150$, $b=80$ , $h=50$,  $d\cong50.8$ , $t=2$ , $L_{1}\cong66.7$, $L_{2}\cong22$,  $w=8$, $a=3$, $N_{c}=54$, $m=2$ | $\beta_{1}, \ldots, \beta_{18}=1$ | $\beta_{19}, \ldots, \beta_{36}\cong0.04$ |
|  |  |  | $\beta_{37}, \ldots, \beta_{54}\cong0.29$ |  |
